# High-density genetic mapping reveals QTLs associated with Huanglongbing tolerance in citrus

**DOI:** 10.3389/fpls.2026.1848541

**Published:** 2026-07-08

**Authors:** Popat Nanaso Gaikwad, Gurupkar Singh Sidhu, Gurpreet Kaur, Harmanpreet Kaur, Manmohan Dhkal, Pooja Manchanda, H. S. Rattanpal, Parveen Chhuneja

**Affiliations:** 1 School of Agricultural Biotechnology, Punjab Agricultural University, Ludhiana, Punjab, India; 2 Gurdev Singh Khush Institute of Genetics, Plant Breeding and Biotechnology, Punjab Agricultural University, Ludhiana, Punjab, India; 3 Department of Plant Pathology, Punjab Agricultural University, Ludhiana, Punjab, India; 4 Department of Fruit Science, Punjab Agricultural University, Ludhiana, Punjab, India

**Keywords:** DdRAD-sequencing, F_1_ population, Huanglongbing (HLB), linkage map, mapping, quantitative trait loci (QTL)

## Abstract

**Introduction:**

Citrus greening, also known as Huanglongbing (HLB) is a devastating disease in citrus that has severely impacted the global citrus industry. Commercially cultivated varieties, including tangerines (‘Daisy’ tangerines, *C. reticulata*), are highly susceptible to HLB, whereas *Poncirus trifoliata* and its hybrids, such as Carrizo citrange, are widely recognized for their tolerance to HLB disease. While there is no effective control currently available, breeding of HLB-resistant varieties remains the most practical, cost-effective and long-term sustainable strategy. Notably, no reports have been documented linking high coverage, marker density, or quantitative trait loci (QTL) to HLB disease tolerance in the Carrizo citrange × “Daisy” tangerine population.

**Methods:**

In current investigation, 120 F_1_ hybrids (intergeneric) derived from “Daisy” tangerine × Carrizo citrange was genotyped using Genotyping-by-Sequencing (ddRAD sequencing), leading to construction of high-density SNP based genetic maps for both parents separately. Using an artificial screening approach, F_1_ individuals were phenotyped for HLB tolerance under insect-protected cage over two consecutive years.

**Results and discussion:**

F_1_ hybrids displayed significant variation in HLB disease-related traits, including disease severity, pH difference, and chlorophyll content. QTL analysis using phenotypic variation from both years identified five QTL clusters, located on Ch2 and Ch6 on Carrizo citrange map and Ch1 and Ch6 on ‘Daisy’ tangerine map. Five regions exhibited co-localization of QTLs associated with two traits. The identified major QTLs explained high phenotypic variation, highlighting significant role in HLB tolerance. This is the high coverage with more marker density were used to identify QTLs associated with HLB tolerance using SNP markers. These findings provide first evidence of quantitative genetic basis of HLB tolerance in Carrizo citrange, while also revealing that ‘Daisy’ tangerine harbors valuable genetic regions and these regions will used to improve HLB tolerance. This information will be valuable for future efforts in breeding, fine-mapping, genetic engineering, and marker-assisted selection aimed at enhancing disease tolerance.

## 1 Introduction

Citrus is the world’s most important fruit crop, grown across more than 114 countries (Yang et al., 2024; Deng et al., 2022; Talon and Gmitter, 2008). Among citrus diseases, such as *Phytophthora*, Alternaria brown spot (ABS), citrus tristeza virus (CTV), citrus nematode, and citrus leprosis virus (CiLV), Huanglongbing (HLB) is the most destructive, posing a severe threat to citrus growers globally (Ramekar et al., 2024; Gaikwad et al., 2023; Albrecht and Bowman, 2012). In India, citrus greening was first reported in the 1960s and has subsequently been reported in various citrus-growing regions, where it was identified as the primary cause of citrus dieback disease (Das et al., 2014). The disease has also rapidly spread across many citrus-producing countries worldwide. Because of its widespread prevalence and inadequate and ineffective management strategies, numerous citrus industries have suffered a steady and significant decline in production (Huang et al., 2018). Since its detection in Florida in 2005, HLB has led to a significant decline in citrus acreage and fruit production, with reductions exceeding 70%–74% (Graham et al., 2020).

HLB-infected trees exhibit distinctive symptoms, including leaves with blotchy mottling, stunted yellow shoots, and gradual canopy and branch dieback as the disease progresses. These symptoms arise from the disruption of photoassimilate transport and nutrient uptake caused by the disease, ultimately leading to tree death (Zheng et al., 2018; Blaustein et al., 2018; Fan et al., 2010; Gaikwad et al., 2025). HLB likely causes changes in leaf pH, with a probable acidification of leaf sap or extracts in infected citrus compared to healthy leaves. HLB infection typically reduces leaf chlorophyll content due to impaired photosynthesis and nutrient deficiencies. The bacterium causes phloem dysfunction, which limits nutrient transport (Chen et al., 2023). HLB is primarily attributed to three different species of *Candidatus*: *C*Las (*Candidatus* Liberibacter asiaticus), *C*Laf (*Candidatus* Liberibacter africanus), and *C*Lam (*Candidatus* Liberibacter americanus). Among these species, *C*Las is one of the most virulent and widespread. In Indian citriculture, only the *C*Las species has been reported (Das et al., 2014). *C*Las is a Gram-negative and heat-tolerant bacterium, found exclusively in the phloem tissue of its plant hosts. *C*Las is transmitted through the sap-sucking Asian citrus psyllid (ACP), also known as *Diaphorina citri* (Huang et al., 2018; Wang et al., 2017; Ramadugu et al., 2016). *C*Las is an obligate and insect-transmitted pathogen. *C*Las infects all citrus species and their hybrids within the *Citrus* genus, and a few of the closely related *Citrus* genera (Huang et al., 2018; Ramadugu et al., 2016). The majority of the citrus germplasm is highly susceptible to HLB, including mandarins, pummelos, grapefruit, and tangerines, particularly the “Daisy” tangerine in Northwestern Indian agroclimatic conditions (Ramekar et al., 2024; Suh et al., 2021; Stover et al., 2016a, b). However, tolerance to HLB has been observed in certain types of rootstock, *viz*., trifoliate orange and its hybrids, including Carrizo citrange (Ramadugu et al., 2016; Albrecht and Bowman, 2012, 2011; Shokrollah et al., 2011). Different HLB tolerance levels have been reported: *Poncirus trifoliata* is considered resistant (Albrecht and Bowman, 2012) and partially resistant (Folimonova et al., 2009); *Microcitrus australasica* and Australian finger lime are considered tolerant (Ramadugu et al., 2016); *Citrus medica* Diamante, *C. sinensis* pineapple sweet orange, and *Citrus reticulata* unnamed mandarin are considered susceptible (Ramadugu et al., 2016); and other susceptible genotypes include: Sweet orange (Pera and Tobias), *Citrus halimii* (Pomeroy, Benecke, Barnes, and Rubidoux), *Atalantia* species (*A. citroides* and *A. ceylanica*), and *Citrus × sinensis* “Valencia”, which was reported by Alves et al. (2021a, b) and Cifuentes-Arenas et al. (2022).

The molecular mechanisms underlying *C*Las pathogenesis remain poorly understood (Ma et al., 2022; Martinelli and Dandekar, 2017). Despite efforts to decipher the genetic basis of resistance or susceptibility to HLB, significant progress has yet to be made. For instance, in India, no such QTLs associated with *C*Las infection or HLB tolerance have been reported. Furthermore, sustainable strategies for managing and controlling HLB in endemic regions remain elusive. Globally, the development of resistant/tolerant varieties is considered the most effective long-term strategy for combating this devastating disease. Studies highlighting variations in tolerance or susceptibility to HLB among citrus species and their relatives offer promising opportunities for breeding and selecting cultivars with improved resistance or tolerance (Huang et al., 2018; Miles et al., 2017; Killiny et al., 2017; Ramadugu et al., 2016; Richardson and Hall, 2013). Conventional breeding approaches, involving the crossing of elite varieties with resistant ones, may help to achieve this goal. Although introgressing resistance traits into elite cultivars often requires multiple rounds of backcrossing to recover desirable commercial traits, it also faces several challenges, such as polyembryony (Sidhu et al., 2024b), rootstock–scion incompatibility, and a long juvenility period. Given that the citrus breeding cycle ranges from 5 to 10 years, the urgency to rescue citrus growers necessitates faster solutions from HLB infestation. Identifying QTLs related to HLB resistance/tolerance can accelerate the development of resistant hybrids through marker-assisted selection (MAS) or genetic engineering (Huang et al., 2018; Albrecht and Bowman, 2012, 2011).

A high-resolution and precise genetic map is essential for identifying QTLs linked to phenotypic traits. Advances in high-throughput sequencing and single-nucleotide polymorphism (SNP) genotyping have enabled the construction and saturation of high-resolution genetic maps using numerous markers at a reasonable cost (Kaur et al., 2022, 2021; Deschamps et al., 2012). While primarily limited to herbaceous plant species, high-resolution genetic mapping has increasingly been applied to woody perennial plants. High-resolution genetic maps have significantly improved for certain citrus species, including a variety of sweet oranges, mandarins, and pummelos through high-throughput genotyping (Singh et al., 2023; Huang et al., 2018; Imai et al., 2017; Curtolo et al., 2017; Yu et al., 2016; Guo et al., 2015; Shimada et al., 2014; Ollitrault et al., 2012a, b; Lyon, 2008). However, compared to well-explored model plants and field crops, citrus research lags in developing high-density genetic maps with precise accuracy because citrus is highly heterozygous (Gaikwad et al., 2026a, b, c; 2024; Tupke et al., 2021), and the development of mapping populations is quite difficult because of its polyembryonic nature (Sidhu et al., 2024a, b). Notably, no high-resolution genetic map has yet been developed for trifoliate orange or its hybrids, including Carrizo citrange.

This study initiates research into HLB disease infestation in citrus hybrids through continuous phenotyping of an F_1_ segregating population and QTL mapping through genotyping by sequencing. The primary goals of this study were to (i) evaluate HLB disease severity in an F_1_ citrus hybrid population derived from a “Daisy” tangerine × Carrizo citrange under intense HLB pressure using the phenotypic traits including percent disease index (PDI), percentage pH difference between pre- and post-HLB infection leaf extracts (pH%), and leaf chlorophyll content (LC); (ii) construct high-density genetic maps of “Daisy” tangerine and Carrizo citrange using ddRAD sequencing; and (iii) identify QTLs associated with HLB-related traits on the constructed genetic maps.

## 2 Materials and methods

### 2.1 Plant material and mapping population development

The tolerant parent, Carrizo citrange (♂), and the susceptible parent “Daisy” tangerine (♀) were utilized for the development of the F_1_ citrus hybrid population. A population of 120 identified hybrids was derived from an intergeneric cross between the “Daisy” tangerine (Fortune mandarin × Fremont mandarin) and the Carrizo citrange [*C. sinensis* “Washington” sweet orange × *P. trifoliata* (L.) Raf.] by hand pollination in March 2021. Hybridization was done on 8- to 10-year-old healthy plants in the College Orchard, Department of Fruit Science, Punjab Agricultural University (PAU), Ludhiana, Punjab, India. The seeds of the harvested fruits were sown in October 2021, and these seedlings were transplanted in August 2022.

### 2.2 Optimization of the artificial HLB screening method

A total of 120 2-year-old F_1_ hybrid seedlings, along with parents, were used for the artificial screening of the HLB disease. In addition, the same-age seedlings of five unifoliate species, namely, Kinkoji (*Citrus obovoidea* Takahashi), Rough lemon (*Citrus jambhiri* Lush), Gou Tou (*Citrus* × *aurantium* cv. Gou Tou L.), Cleopatra (*Citrus reshni* Hort. ex Tanaka), and Volkamer lemon (*Citrus volkameriana* Osbeck), and two trifoliate hybrids, Swingle citrumelo (Duncan grapefruit × *P. trifoliata*, × *Citroncirus* spp. RUTACEAE), and X639 (Cleopatra mandarin × *P. trifoliata* (L.) Raf., × *Citroncirus* spp. RUTACEAE) were used as controls with seven replicates of each for the HLB phenotyping. For the artificial screening, 2-year-old symptom-based positive plants, *viz*., “Daisy” tangerine (80 plants) and Mosambi [*C. sinensis* (L.) Osbeck] (70 plants) budded on Rough lemon, were collected. Out of the total plants collected, 62 “Daisy” tangerine and 58 Mosambi plants tested positive for the presence of *C*Las, confirmed through polymerase chain reaction (PCR) using gene-specific primers (**Supplementary File S1**). All 120 F_1_ hybrid plants and the 120 plants that tested positive (“Daisy” tangerine and Mosambi) were shifted into an insect-proof cage with 40-mesh screening at the School of Agricultural Biotechnology, PAU, Ludhiana. Before they were moved into the cage, all 120 F_1_ hybrids and their parents were tested for *C*Las, and all were found to be negative (**Supplementary Figure 1**). During screening, the daily temperature varied from 20 °C to 38 °C, the relative humidity ranged from 25% to 40%, and the illumination was natural.

All F_1_ hybrid plants and the 120 plants that tested positive were arranged in a specific order: one F_1_ seedling followed alternately by one plant that tested positive. After repeating the arrangement of one F_1_ seedling and one positive plant five times, one control plant was placed (**Supplementary Figure 2**). Adult insects were collected using a 2.5-L bottle aspirator from the College Orchard, Department of Fruit Science, PAU, Ludhiana. Thereafter, force feeding of the collected insects was done on the twigs that tested positive for 24–36 h in the bottle aspirator itself. After force feeding, the insects were released from eight directions so that they could spread uniformly in the cage (**Supplementary Figure 2**). A total of ~28,800 insects were released into the cage for HLB disease evaluation. All plants were maintained as per the package of practices for the cultivation of fruits given by the PAU, Ludhiana. No pesticides were sprayed throughout the whole experiment to maintain the psyllid population, proper feeding, and colonization of *C*Las.

### 2.3 Detection and quantification of *C*Las titer through qRT-PCR assay

The provided artificial HLB disease pressure was high, which showed homogeneous inoculation of *C*Las inside the cage. Before the HLB disease evaluation, *C*Las titer quantification of each F_1_ hybrid seedling, along with control varieties, was checked through a multiplex real-time PCR method (Applied Biosystems™ StepOne RT-PCR, USA) using a 16S ribosomal gene probe (Ramadugu et al., 2016), with COX used as an internal control. Fully mature leaves (5 to 10) were selected from each seedling quadrant for DNA and RNA isolation. The petioles and midribs were removed from selected leaves, which were then cut into small pieces. These small pieces (midrib and petiole tissues, 100 mg) were used for DNA isolation using a modified CTAB (cetyl trimethyl ammonium bromide) method (Cheng et al., 2003) and RNA isolation using the TRIzol method (RNAiso Plus). The quality and integrity of the DNA and RNA were assessed using a 0.8% agarose gel, while the quantity was determined with a Thermo Scientific NanoDrop™ 1000 spectrophotometer (Wilmington, US). The reaction mixture included 1× SYBR green master mix, 0.5 µL of forward and reverse primer (5 µM), and 100 ng of cDNA. Ct values (cycle threshold) from qPCR for *C*Las detection and quantification were normalized.

### 2.4 Phenotypic screening and HLB disease evaluation

All 120 F_1_ seedlings, along with control plants, were phenotyped for HLB disease-related traits, including PDI, pH%, and LC. Evaluations of HLB disease-related traits were performed two times per year from 2023 to 2024 in September and October because these months were considered an optimal time for HLB disease evaluation. HLB disease symptoms were most evident during these two months. The averaged evaluation results from both years were used for mapping. During each disease screening period, symptoms were visually assessed twice per seedling on both sides.

The HLB disease severity was assessed on a 0–9-point scale according to Pustika et al. (2008) with minor modifications: 0 = asymptomatic leaves; 1 = <50% mild blotchy leaf pattern; 2 = >50% blotchy leaf pattern; 3 = <50% symptomatic leaves with interveinal chlorosis; 4 = >50% symptomatic leaves with interveinal chlorosis; 5 = <50% symptomatic leaves with interveinal chlorosis, and vein corking; 6 = >50% symptomatic leaves with interveinal chlorosis, and vein corking; 7 = <50% leaves abscised and branch showing dieback; 8 = >50% leaves abscised and branch showing dieback; 9 = branch desiccated and all leaves abscised. The PDI was calculated for 10 leaves per F_1_ hybrid using the following formula (McKinney, 1923):


$$\text{Percent Disease Index }(\text{PDI})=\sum \frac{\left(s\;\times n\right)}{\left(S\;\times N\right)}\times 100$$

where *s* is the disease grade, *n* is the number of leaves in the disease grade, *S* is the highest disease rating scale, and *N* is the total number of hybrids.

For the pH analysis, the leaves of each hybrid seedling were macerated in 95% ethanol, and the prepared extract was kept at room temperature for 48 h. The residual solvent was removed in a BOD incubator at 45 °C for 15 min. The extracted samples were centrifuged at 15,000 rpm for 20 min. Then, the supernatant was collected, and impurities were discarded. These crude extracts were used to check the pH of each sample with the help of a digital pH meter (Tachakittirungrod et al., 2007). The pH was checked before and after *C*Las infection in F_1_ hybrid seedlings, the parents, and the controls. The percent difference in pH was calculated between before and after *C*Las infection.

Leaf chlorophyll content was measured using an SPAD meter (Apogee Instruments, Inc., USA) with five replications of five leaves of each F_1_ hybrid seedling after CLas infection, and then the average was calculated.

### 2.5 Descriptive statistical analysis

All phenotypic data were statistically analyzed using IBM SPSS Statistics 22.0 (Statistical Package for the Social Sciences). The normality of the phenotypic traits was evaluated using the Shapiro–Wilk test. Traits exhibiting deviation from normality were transformed prior to statistical analysis. Arcsine square-root transformation improved the distribution of the HLB severity trait, whereas logarithmic transformation was applied to pH and LC traits. Subsequent analyses were conducted using the transformed data, as appropriate. A Student’s *t*-test was performed to compare the phenotypic traits of unifoliate and trifoliate genotypes, including Ct value, HLB disease severity, pH, and LC, with statistical significance determined at *p* ≤ 0.05. Histograms for each disease-related trait were generated based on the mean scores separately for both years and the average of both years. Pearson’s correlation coefficients were computed between years using their mean values for each disease-related trait and its average using RStudio.

### 2.6 DNA extraction, construction of library, and ddRAD-sequencing

DNA was extracted from fully mature leaf tissue (100 mg) of F_1_ seedlings and their parents using the modified CTAB method (Cheng et al., 2003). The quality and integrity of the DNA were assessed using a 0.8% agarose gel, while the quantity was determined with a Thermo Scientific NanoDrop™ 1000 spectrophotometer (Wilmington, USA). All samples were normalized to ~300 ng/µL and utilized for ddRAD-seq analysis. DNA digestion, adapter ligation, library construction, and sequencing were performed by NGB Diagnostics Pvt. Ltd., New Delhi, India. For the citrus ddRAD library preparation, the DNA was double-digested using rare-cutting (EcoRI-HF) and frequent-cutting (MseI) enzymes. EcoRI-specific P1 adapters were used to ligate the digested DNA fragments, and another adapter, MseI-specific P2, was ligated using a T4 ligase enzyme (New England BioLabs). The prepared DNA library was sequenced using the Illumina HiSeq™ X10 platform (Illumina^®^ Inc., San Diego, CA, USA) on a single lane via V4 sequencing chemistry.

### 2.7 Identification and genotyping of single-nucleotide polymorphisms

FASTQ files containing paired-end reads and low-quality reads and adaptors were filtered out using Trimmomatic tools (Bolger et al., 2014). Reads shorter than 50 base pairs were excluded. Then, the cleaned reads were aligned to the *C. reticulata* (reference genome) chromosome-level genome assembly (GCA_036169575.1) using Bowtie 2 with default parameters. Then, the Sequence Alignment Map (SAM) files were converted to Binary Alignment Map (BAM) format and subsequently sorted through BAMtools. SNP calling was achieved through the “*mpileup*” command in BCFtools. Then, the obtained SNPs were filtered to exclude those with a minimum read depth of less than 4, a base quality score below 20, or a minor allele frequency (MAF) ≤ 0.05.

### 2.8 Parental genetic map construction

Segregating SNP markers of types “lm × ll”, “nn × np”, and “hk × hk” were utilized to construct the linkage map following the pseudo-testcross strategy, suitable for outbreeding species (Grattapaglia and Sederoff, 1994; Van-Ooijen, 2011), implemented in Lep-MAP3 (v0.5) (Rastas, 2017). The resulting SNPs were initially assigned to chromosomes based on their physical positions in the *C. reticulata* reference genome. Recombination frequencies (RFs) and logarithms of odds (LOD) scores were calculated separately for each parent. To reduce redundancy and improve map quality, binning was performed by (1) merging identical markers (100% similarity) and retaining only the first marker within each group, and (2) selecting markers with the fewest missing data across individuals (Vision et al., 2000). The genetic positions of the bin markers were determined using the complete marker dataset, and these bin markers were then used to construct a high-resolution linkage map. Map distance was calculated in cM (centiMorgans) through the algorithm of regression mapping and the Kosambi mapping function. The numbering of chromosomes, marker names, and SNP positions was based on the reference genome information. The final linkage maps were visualized using MapChart v2.32 (Voorrips, 2002).

### 2.9 Quantitative trait locus analysis

Quantitative trait locus (QTL) mapping was conducted using the R/qtl software package (v1.50) (Broman et al., 2003; Arends et al., 2010) based on data derived from 120 F_1_ hybrids. In R/qtl, composite interval mapping (CIM) was performed using the cim() function, with an LOD threshold of 2.5–3 (Zeng, 1994). QTL peak positions were identified based on the maximum LOD score. Multiple QTL models were developed using the makeqtl() and fitqtl() functions to estimate the percentage of phenotypic variance explained (PVE) by each QTL. In cases where a single QTL was detected, its position was further refined using the refineqtl() and makeqtl() functions. Additional QTLs were identified using addqtl() within the multiple-QTL framework, and potential interactions between QTLs were assessed with the addint() function. Final estimates of phenotypic variance (PV) for the detected QTLs were obtained using the fitqtl() function.

Morphological data were analyzed independently for each trait and each year, as well as for the average across both years. We used different LOD thresholds for the female and male parental maps based on the stability and strength of the detected QTLs across seasons. In the female parent, several consistent QTLs with comparatively high PVE values were repeatedly detected at LOD values above 2.5 across different seasons and traits. In contrast, in the male parent, reliable and stable QTLs were primarily observed above the 3.0 LOD threshold. A PV exceeding 10% was considered to be a putative QTL for HLB tolerance and other disease-related traits. The nomenclature for each QTL followed a specific format: for example, in “*qHLB-Dy.pau1.1*”, “*qHLB*” indicates a QTL associated with tolerance to HLB disease and related traits, “*Dy*” refers to the “Daisy” tangerine parent, “*pau*” represents the university name, and “*1.1*” denotes the first QTL for the particular trait located on chromosome 1.

## 3 Results

### 3.1 Phenotyping of the F_1_ hybrid population for HLB disease-related traits

**Figure 1:**
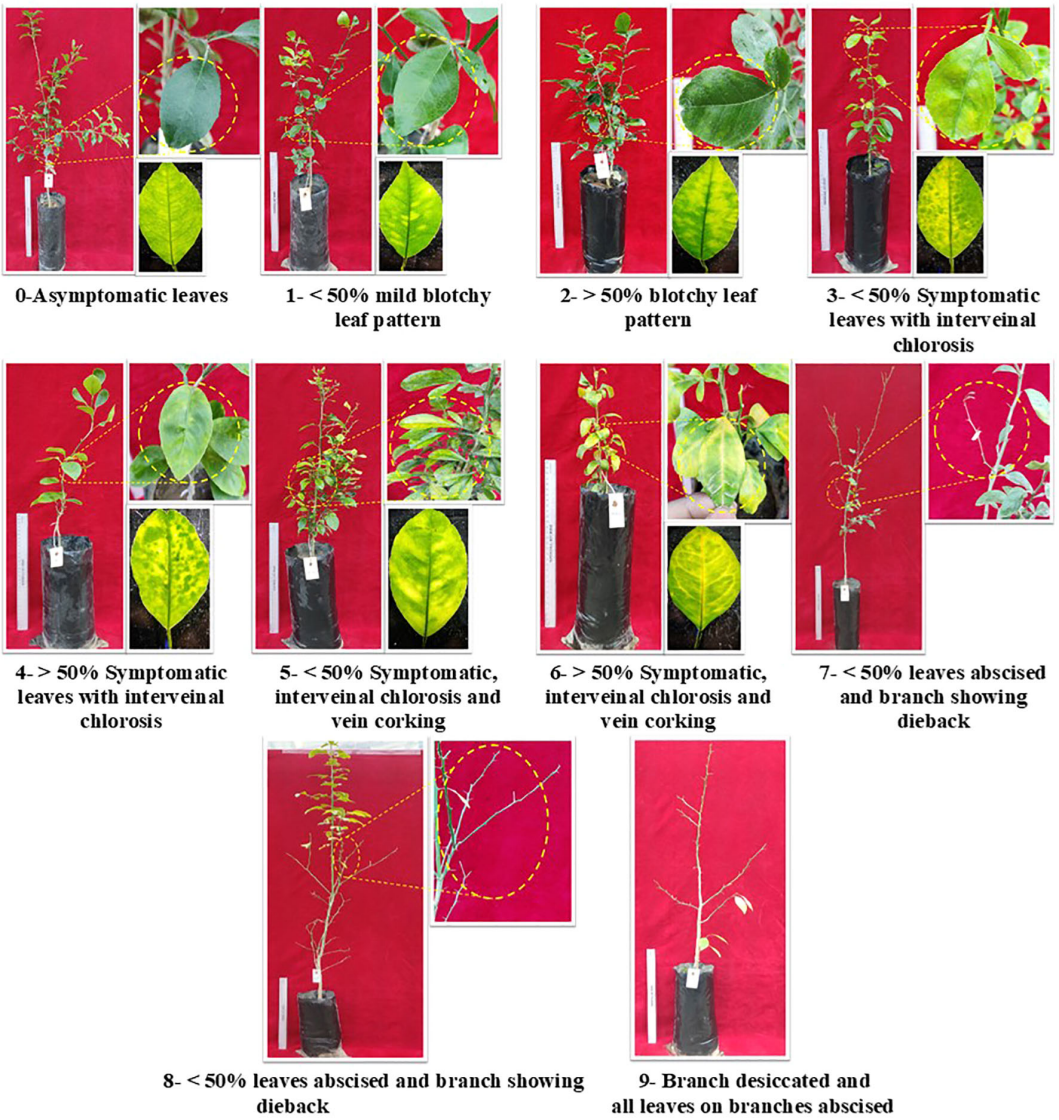
Disease score (0–9) for HLB phenotypic screening in the F_1_ hybrid population derived from the crossing of “Daisy” tangerine (♀) and Carrizo citrange (♂).

**Figure 2:**
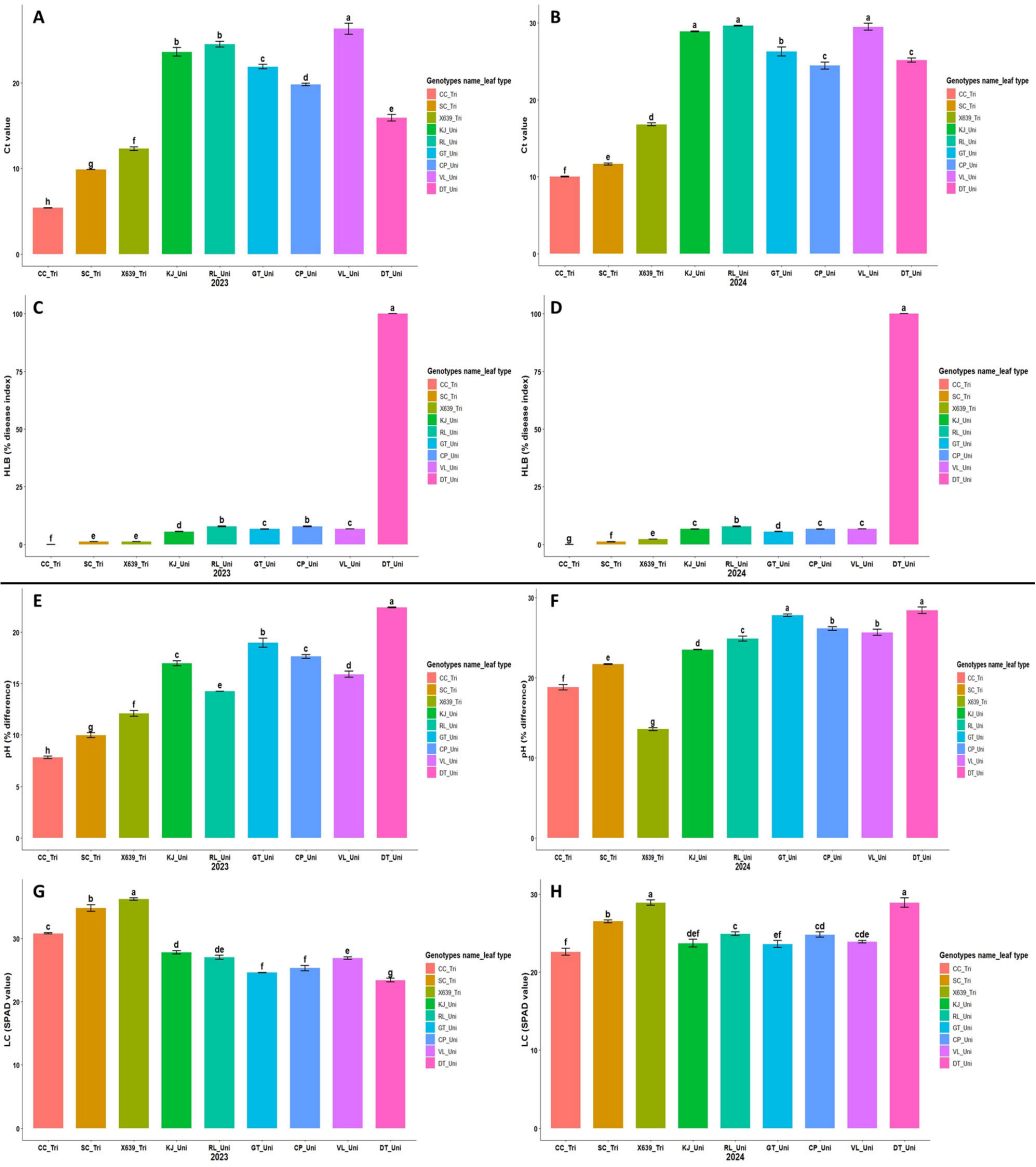
Detection of *Candidatus* Liberibacter asiaticus (*C*Las) infection through qRT-PCR and evaluation of Huanglongbing (HLB)-associated disease traits among citrus control varieties in 2023 and 2024. **(A, B)** represent the cycle threshold (Ct) values obtained through qRT-PCR analysis in 2023 and 2024, respectively. **(C, D)** show the HLB disease index values recorded in 2023 and 2024, respectively. **(E, F)** indicate the pH (%) difference, while **(G, H)** represent the leaf chlorophyll content (SPAD values) in 2023 and 2024, respectively. The trifoliate hybrids included Carrizo citrange (CC_Tri), Swingle citrumelo (SC_Tri), and X639 (X639_Tri), whereas Kinkoji (KJ_Uni), Rough lemon (RL_Uni), Gou Tou (GT_Uni), Cleopatra (CP_Uni), Volkamer lemon (VL_Uni), and Daisy tangerine (DT_Uni) were categorized as unifoliate varieties. Data are presented as mean ± standard error (SE). Different lowercase letters above the bars indicate statistically significant differences among genotypes at *p* ≤ 0.05 according to the applied *post hoc* comparison test. HLB, Huanglongbing; CLas, Candidatus Liberibacter asiaticus; Ct value, cycle threshold value; LC, leaf chlorophyll content measured using a SPAD meter.

A population consisting of 120 F_1_ hybrids, along with five unifoliate and two trifoliate species with seven replicates, was used for HLB phenotyping. HLB disease symptoms appeared during September and October in both years. Symptoms such as blotchy, yellow, asymmetrical mottling of the leaves, interveinal chlorosis, vein corking, leaves abscised, branch dieback, and branch desiccation were observed (**Figure 1**). After exposure to HLB disease pressure inside the cage, all the phenotyped seedlings were tested for *C*Las colonization using multiplex real-time PCR during the disease evaluation period. All F_1_ hybrids along with the unifoliate and trifoliate controls were infected by *C*Las in both years (**Figure 2**). *C*Las presence was confirmed in both the F_1_ hybrid population and the controls. The entire F_1_ hybrid population, along with the controls, had high titers of the *C*Las bacterium (Ct value of *C*Las ranged from 18.5 to 31.5). The results indicate that all F_1_ hybrids and controls were sufficiently inoculated with the *C*Las bacterium under HLB pressure inside the cage and that all F_1_ hybrids, including both parents, tested positive (**Supplementary Figure 3**).

**Figure 3:**
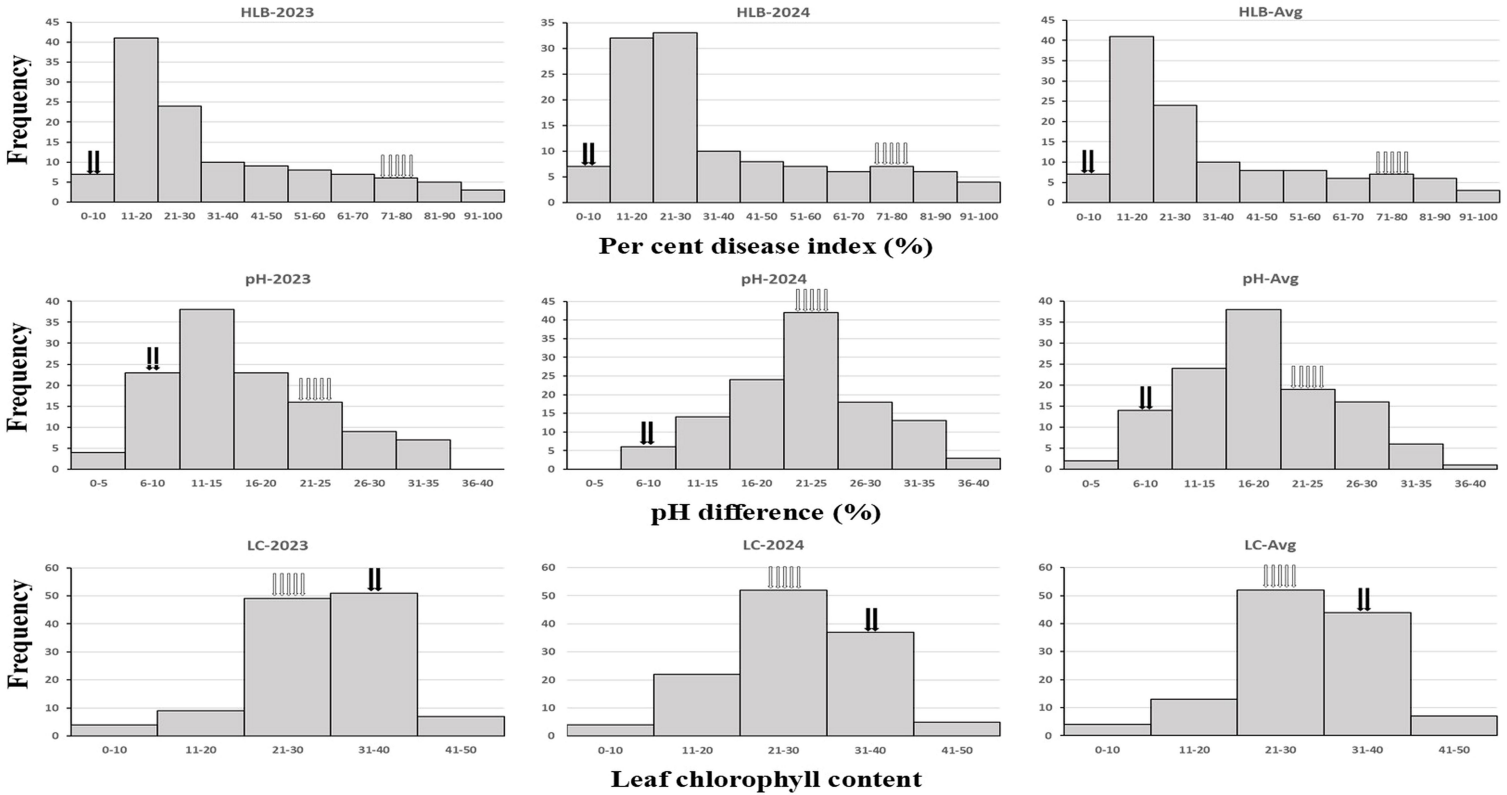
Frequency distributions of percent disease index (HLB) (%), pH difference (%), and leaf chlorophyll content in the F_1_ hybrid population over 2 years and their average. The blank arrows indicate Swingle citrumelo and X639, whereas the filled arrows indicate Kinkoji, Rough lemon, Gou Tou, Cleopatra, and Volkamer lemon.

**Figure 4:**
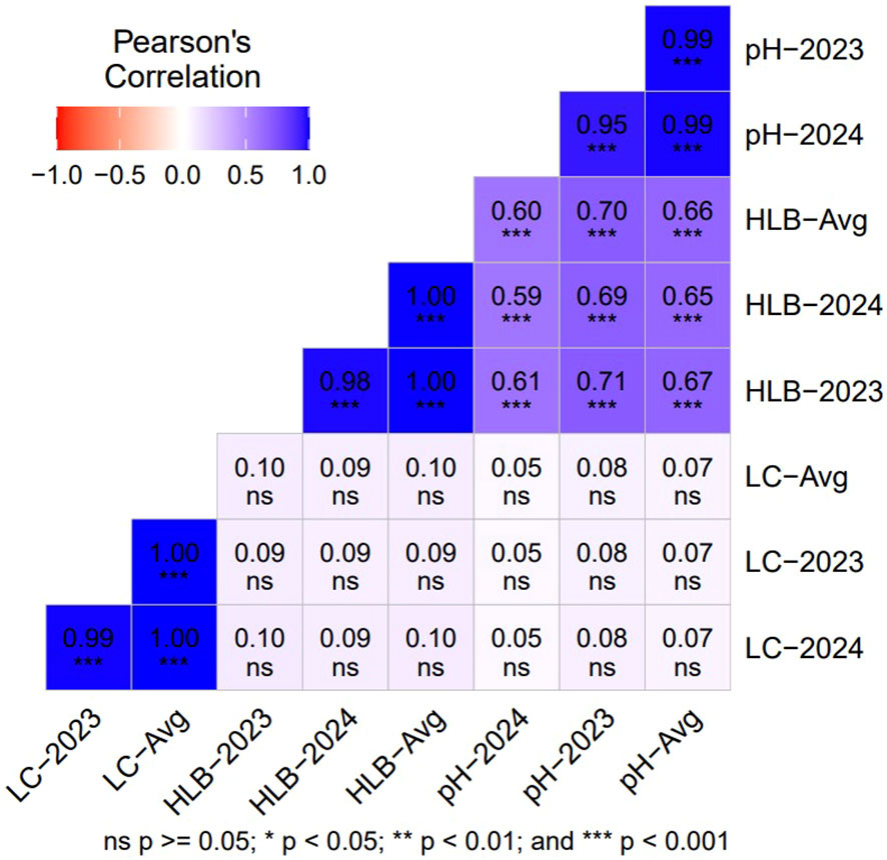
Pearson’s correlation coefficients (*r*) between nine HLB disease-related traits [HLB-2023 (Y1—year 1), HLB-2024 (Y2—year 2), HLB-AVG (average of both years), pH-2023 (Y1—year 1), pH-2024 (Y2—year 2), pH-AVG (average of both years), LC-2023 (Y1—year 1), LC-2024 (Y2—year 2), and LC-AVG (average of both years)].

The HLB disease evaluation of the control varieties was carried out in 2023 and 2024 for HLB disease-related traits, including PDI, pH, and LC, and was recorded (**Figure 2**). PDI and pH values in trifoliate species ranged from 1.1% to 2.2% and from 10.0% to 21.7%, respectively, which were significantly lower than those observed in unifoliate species, where the values ranged from 5.6% to 7.8% and 14.3% to 27.8%, respectively, at *p* ≤ 0.05. In contrast, LC values in trifoliate species ranged from 26.5 to 36.2, which were higher than those recorded in unifoliate species (23.6–27.8) among the control genotypes at *p* ≤ 0.05. The PDI, pH, and LC values in the “Daisy” tangerine ranged from 100%, 22.4% to 28.4%, and 23.4% to 28.9%, respectively, whereas in the Carrizo citrange, the corresponding values ranged from 0.0%, 7.8% to 18.8%, and 22.6% to 30.6%, respectively. In the F_1_ hybrid population, the PDI and pH were significantly higher in 2024 (33.7% and 22.5%, respectively) than in 2023 (32.0% and 16.0%, respectively). In the case of leaf chlorophyll content, it decreased from 28.8 (2023) to 26.8 (2024). The frequency distributions of PDI, pH, and LC in the F_1_ hybrid population are presented in **Figure 3**. According to the frequency distribution, the quantitative variation of HLB disease-related traits was recorded in both years (2023 and 2024) and in the average of both years. Pearson’s correlation coefficients (*r*) between HLB disease-related traits were determined (each trait with 2-year data and their average). The highly positive correlations observed between 2023 and 2024 were 0.9, 0.9, and 0.9 for the PDI, pH, and LC traits, respectively. The positive correlation recorded between PDI and pH for both years ranged from 0.6 to 0.7. Lower correlation coefficients were recorded between LC and pH, and between LC and PDI traits (**Figure 4**). A broad and continuous range of variation was observed among the F_1_ hybrids for all HLB-related traits, suggesting quantitative inheritance and differential responses to HLB infection.

In the F_1_ hybrid population, the mean PDI score ranged from 0.0 to 100.0 across both years, with coefficients of variation (CVs) of 83.1%, 81.1%, and 81.7% for 2023, 2024, and the average of both years, respectively (**Table 1**). The mean pH score ranged from 1.6% to 38.2% for both years with CVs of 47.2%, 30.4%, and 36.6% for 2023, 2024, and the AVG of both years, respectively. The mean LC score ranged from 4.0% to 46.6% for both years, with CVs of 28.8%, 26.8%, and 27.8% for 2023, 2024, and the AVG of both years, respectively. Furthermore, kurtosis and skewness values close to or below one for all evaluated traits suggested continuous variation and an approximately normal distribution, supporting the suitability of the population for QTL mapping studies (**Table 1**).

**Table 1:** Descriptive statistical results of phenotypic data for the F_1_ citrus population derived from the cross of “Daisy” tangerine (♀) with Carrizo citrange (♂).

Trait name	Min	Max	Range	Mean ± SE	Coefficient of variance (%)	Skewness	Kurtosis
HLB-2023	0.0	100.0	100.0	32.0 ± 2.4	83.1	1.1	−0.1
HLB-2024	0.0	100.0	100.0	33.7 ± 2.5	81.1	1.0	−0.1
HLB-Avg	0.0	100.0	100.0	32.9 ± 2.5	81.7	1.0	−0.2
pH-2023	1.6	35.3	33.7	16.0 ± 0.7	47.2	0.7	−0.1
pH-2024	7.8	38.2	30.4	22.5 ± 0.6	30.4	0.1	−0.4
pH-Avg	5.5	36.8	31.3	19.2 ± 0.6	36.6	0.4	−0.3
LC-2023	4.0	46.6	42.6	28.8 ± 0.8	28.5	-0.4	0.5
LC-2024	4.0	45.2	41.2	26.8 ± 0.7	29.2	-0.4	0.4
LC-Avg	4.0	45.9	41.9	27.8 ± 0.7	28.8	-0.4	0.5

### 3.2 High-throughput ddRAD sequencing and SNP genotyping

**Figure 5:**
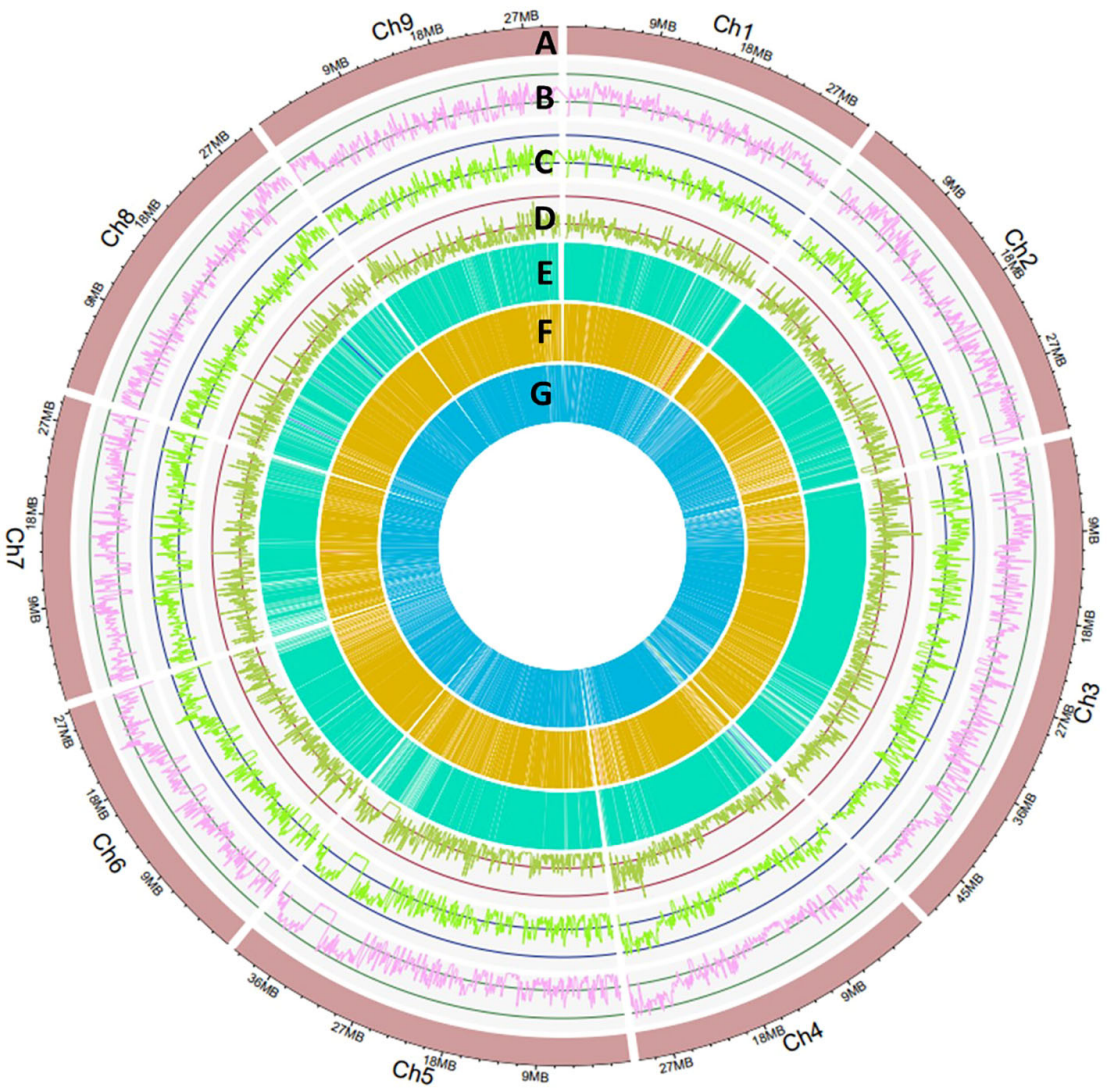
Distribution of SNPs identified in the F_1_ hybrid population from “Daisy” tangerine (♀) × Carrizo citrange (♂) in citrus. Track **(A)** represents 9 chromosomes of different lengths. Track **(B)** shows the total number of SNPs obtained from each chromosome, track **(C)** depicts the SNPs retained after removing indels, track **(D)** shows the SNPs retained after missing data filtering, track **(E)** represents the mapped SNPs, track **(F)** shows the total number of unique mapped loci on the female chromosomes, and track **(G)** shows the total number of unique mapped loci on the male chromosomes.

All 120 F_1_ hybrids and the two parental genotypes (“Daisy” tangerine and Carrizo citrange) were sequenced using the ddRAD Illumina HiSeqTM X10 platform and generated 234.8 million paired-end (PE) reads that had a total length of 150 bp with a sequencing depth of 0.5×. All F_1_ hybrid populations and parental genotypes exhibited adequate quality of sequenced reads. The “Daisy” tangerine and Carrizo citrange parents had average reads of 3.4 and 1.7 million, respectively. The average number of reads for the 120 F_1_ hybrids ranged from 0.6 to 9.2 million reads, with an average of 2.0 million reads (**Supplementary Figure 4**). The cleaned reads were aligned to the reference genome with a 69.3%–84.3% alignment percentage, with an average of 79.3%. A total of 84,554 putative SNPs were identified. These SNPs were filtered for 5% MAF and 25% missing data, out of which the female linkage map had 42,277 SNPs, whereas the male linkage map had 42,277 SNPs (**Tables 2**, **3**). After rigorous filtering of chi-square (χ²) at *p* = 0.001, the resulting high-quality SNPs were retained with marker configuration codes of “lm × ll”, “nn × np”, and “hk × hk” and utilized to construct genetic linkage maps. A total of 1,117 unique loci were mapped on Carrizo citrange and 788 unique loci on “Daisy” tangerine (**Figure 5**). These identified SNPs were utilized for the construction of a linkage map for both parents separately.

**Table 2:** Summary statistics of the “Daisy” tangerine genetic linkage map, constructed using the F_1_ hybrid population derived from the cross of “Daisy” tangerine (♀) and Carrizo citrange (♂).

Chr. no.	Number of SNPs	No. of loci	SNP/locus	Length (cM)	Avg. spacing (cM)	Max. spacing (cM)
1	4,925	87	56.6	110.8	1.3	7.5
2	6,973	135	51.7	156.7	1.2	16.7
3	6,912	115	60.1	115.0	1.0	5.0
4	5,406	105	51.5	136.7	1.3	19.2
5	5,502	107	51.4	123.3	1.2	9.2
6	4,240	78	54.4	125.8	1.6	34.2
7	4,947	87	56.9	141.7	1.6	13.3
8	1,548	38	40.7	37.5	1.0	4.2
9	1,824	36	50.7	48.3	1.4	14.2
Total	42,277	788	52.7	995.8	1.2	13.7

**Table 3:** Summary statistics of the Carrizo citrange genetic linkage map, constructed using the F_1_ hybrid population derived from the cross of “Daisy” tangerine (♀) and Carrizo citrange (♂).

Chr. no	Number of SNPs	No. of loci	SNP/locus	Length (cM)	Avg. spacing (cM)	Max. spacing (cM)
1	4,925	118	41.7	105.0	0.9	2.5
2	6,973	149	46.8	142.5	1.0	5.0
3	6,912	199	34.7	207.5	1.0	7.5
4	5,406	121	44.7	117.5	1.0	8.3
5	5,502	140	39.3	135.0	1.0	8.3
6	4,240	136	31.2	133.3	1.0	5.0
7	4,947	142	34.8	124.2	0.9	2.5
8	1,548	72	21.5	88.3	1.2	21.7
9	1,824	40	45.6	42.5	1.1	4.2
Total	42,277	1,117	37.8	1,095.8	1.0	7.2

### 3.3 Development of high-density parental linkage maps

**Figure 6:**
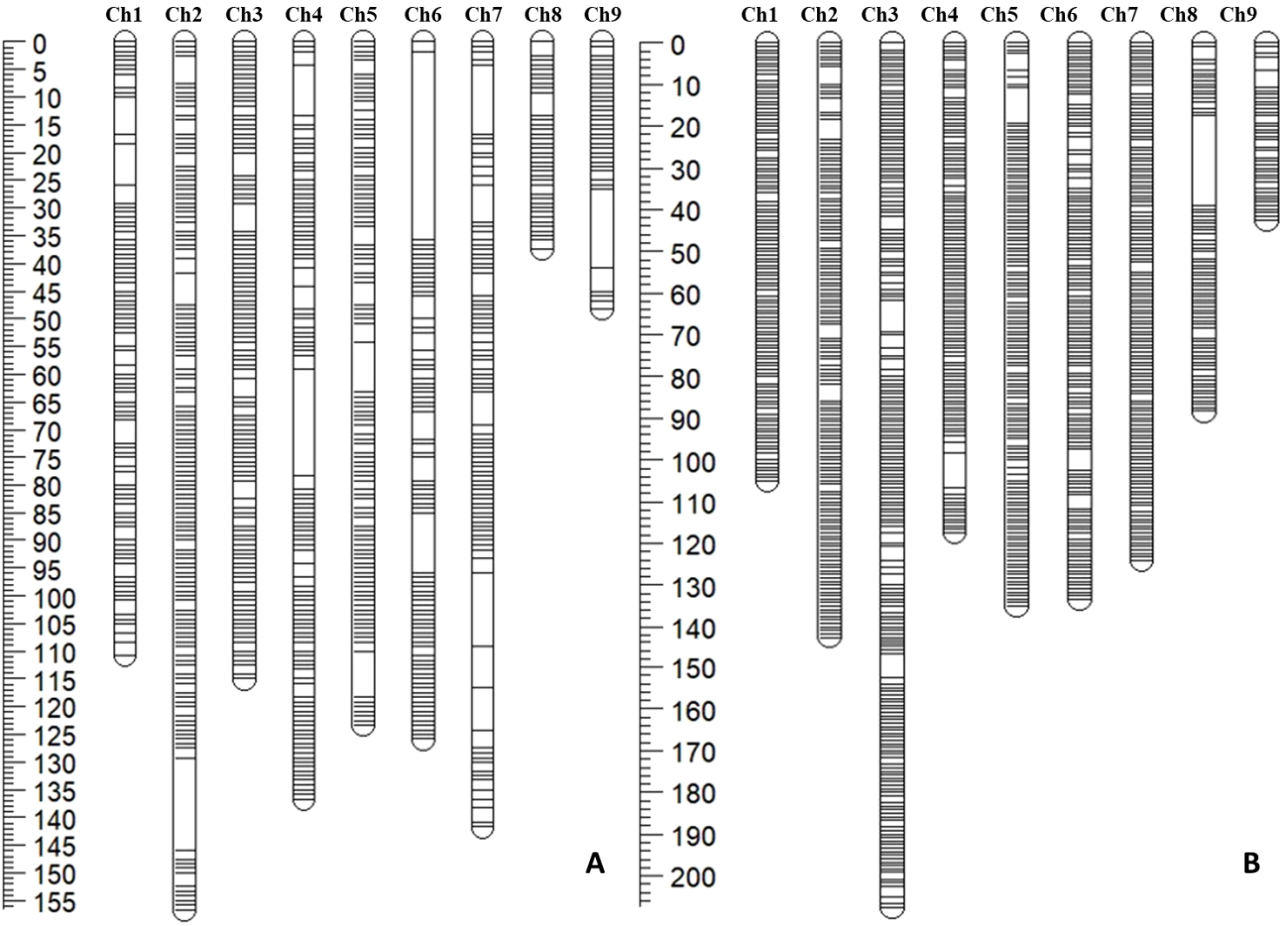
Genetic linkage map of “Daisy” tangerine **(A)**, and Carrizo citrange **(B)** map distances in centiMorgans (cM) are shown on the left side of each genetic map.

For both the male (Carrizo citrange) and female (“Daisy” tangerine) parents, SNPs were clustered under an LOD score of 3.0 to 22.0 into nine chromosomes in both parents. Furthermore, all chromosomes in both the male and female parents preserved their integrity even at an LOD score of 30. The number of chromosomes identified was the same as the haploid chromosome count in the citrus genome. The chromosomes were numbered according to their respective number in the reference genome. For the “Daisy” tangerine, a total of 788 high-quality SNPs were mapped across nine chromosomes, covering a total genetic distance of 995.8 cM, with an average spacing of 1.2 cM between loci (**Table 2**, **Figure 6A**). The number of markers per chromosome varied from 38 in Ch9 to 135 in Ch2. The genetic distances covered ranged from 37.5 cM in Ch8 to 156.7 cM in Ch2. The smallest inter-locus gap on the genome-wide genetic map was 4.2 cM (Ch8), while the largest gap did not exceed 34.2 cM (Ch6). In Ch6, a relatively large gap was observed; however, this was primarily due to the lower number of polymorphic SNP markers available in this specific genomic region. In the Carrizo citrange, 1,117 high-quality SNPs were successfully mapped across nine chromosomes. These chromosomes covered a total genetic length of 1,095.8 cM, with an average distance of 1.0 cM between loci and an average maximum spacing of 7.2 cM (**Table 3**, **Figure 6B**). The number of markers within each chromosome varied in number, with Ch9 containing 40 markers and Ch3 having the highest count at 199. The genetic distances covered ranged from 42.5 cM in Ch9 to 207.5 cM in Ch3. The genome-wide genetic map showed inter-locus gaps ranging from a minimum of 2.5 cM (Ch1 and Ch7) to a maximum of 21.7 cM (Ch8).

### 3.4 Detection of quantitative trait loci linked to HLB disease resistance

The two separate parental genetic maps were developed through the genotyping of SNP markers, and QTLs associated with the three disease-related traits were detected for 2023, 2024, and the average of both years using the R/qtl software.

#### 3.4.1 QTLs identified on the “Daisy” tangerine genetic map

A total of four QTLs associated with HLB disease tolerance were identified on Ch1 and Ch6 for the 2 years and their average on the “Daisy” tangerine genetic map. For QTL mapping in the “Daisy” tangerine, an LOD threshold of 2.5 was selected because several potential QTL peaks were detected close to this value. The QTL on Ch1 was *qHLB-Dy.pau1.1*, located at 39.2 cM in 2023, having an LOD score of 3.6 and a PVE of 9.3%. A similar QTL (*qHLB-Dy.pau1.2*) was found in 2024 at nearly the same position (39.2 cM), indicating its stability over time with an LOD score of 2.7 and a PVE of 8.1%. Additionally, *qHLB-Dy.pau6.1* was detected on Ch6 in 2024, highlighting the presence of another region contributing to HLB tolerance: this was found to be located at 76.0 cM in 2024, having an LOD score of 2.7 and a PVE of 7.2%. The averaged QTL (*qHLB-Dy.pau1.3*) reinforces the consistency of the Ch1 QTL at a similar position, with a LOD score of 2.5 and a PVE of 8.9% (**Table 4**; **Supplementary Figure 5**). Apart from these high LOD scores, other QTLs were detected but were ignored due to their very low PVE percentages.

**Table 4:** Quantitative trait loci identified for HLB disease tolerance on the “Daisy” tangerine (♀) genetic map.

Trait name	QTL name	Year	Chr. no.	Position	LOD	PVE (%)	Marker interval	Confidence interval (cM)
HLB	*qHLB-Dy.pau1.1*	Y1	1	39.2	3.6	9.3	C1_7100290–C1_5611296	38.3–40.0
	*qHLB-Dy.pau1.2*	Y2	1	39.2	2.7	8.1	C1_7100290–C1_5611296	38.3–40.0
	*qHLB-Dy.pau6.1*	Y2	6	76.0	2.7	7.2	C6_21840150–C6_26972654	75.0–79.2
	*qHLB-Dy.pau1.3*	AVG	1	39.2	2.5	8.9	C1_7100290–C1_5611296	38.3–40.0
pH	*qpH-Dy.pau1.1*	Y1	1	39.2	2.9	10.5	C1_7100290–C1_5611296	38.3–40.0
	*qpH-Dy.pau2.1*	Y1	2	136.0	2.8	4.8	C2_7996362–C2_16202004	129.2–145.8
	*qpH-Dy.pau1.2*	AVG	1	37.5	2.9	9.7	C1_15690195–C1_7100290	36.7–38.3
	*qpH-Dy.pau6.1*	AVG	6	74.2	2.9	9.6	C6_25640922–C6_21840150	74.2–75.0
LC	*qLC-Dy.pau2.1*	Y1	2	10.8	2.8	9.0	C2_29248672–C2_29376289	10.0–11.7
	*qLC-Dy.pau4.1*	Y1	4	38.0	2.8	0.8	C4_29580046–C4_28678811	37.5–38.3
	*qLC-Dy.pau2.2*	Y2	2	10.8	2.7	10.3	C2_29248672–C2_29376289	10.0–11.7
	*qLC-Dy.pau4.2*	Y2	4	32.5	2.6	4.9	C4_29155833–C4_29856533	31.7–33.3
	*qLC-Dy.pau2.3*	AVG	2	10.8	2.7	10.1	C2_29248672–C2_29376289	10.0–11.7
	*qLC-Dy.pau4.3*	AVG	4	32.5	2.5	4.8	C4_29155833–C4_29856533	31.7–33.3

HLB, Huanglongbing; pH, percent difference before and after infection; LC, leaf chlorophyll content; Y1, year 2023; Y2, year 2024; AVG, average from both years; LOD, logarithm of odds; PVE, phenotypic variance explained.

Four QTLs were identified for the pH trait on the “Daisy” tangerine genetic map, located on chromosomes 1, 2, and 6. On chromosome 1, *qpH-Dy.pau1.1* was identified at 39.2 cM, with significant effects in 2023, contributing a high PVE of 10.5% with an LOD score of 2.9. The QTL, namely, *qpH-Dy.pau2.1*, recorded a lower PVE (4.8%) and had an LOD score of 2.8. On chromosome 1, *qpH-Dy.pau1.2* was identified in the average of 2023 and 2024 at 37.5 cM, having an LOD score of 2.9 with a PVE of 9.7%. Another QTL was detected in the average of 2023 and 2024, *qpH-Dy.pau6.1*, located on chromosome 6, having a PVE of 9.6% with an LOD score of 2.9 (**Table 4**; **Supplementary Figure 5**).

For leaf chlorophyll content on the “Daisy” tangerine genetic map, six QTLs were found across Ch2 and Ch4. Notably, *qLC-Dy.pau2.1*, *qLC-Dy.pau2.2*, and *qLC-Dy.pau2.3* all mapped to the same position (10.8 cM) on Ch2, across 2023, 2024, and the AVG of both years, respectively, with PVE values ranging from 9.0% to 10.3%, highlighting a stable QTL region. Similarly, three QTLs on Ch4 (*qLC-Dy.pau4.1*, *qLC-Dy.pau4.2*, and *qLC-Dy.pau4.3*) were located between 32.5 and 38.0 cM, although with lower PVE values, suggesting a minor effect of the loci (**Table 4**; **Supplementary Figure 5**). Consistent marker intervals and overlapping confidence intervals across years and traits suggest reliable QTLs, particularly on Ch1 for HLB and pH, and on Ch2 for LC.

#### 3.4.2 QTLs identified on the Carrizo citrange genetic map

Five QTLs associated with HLB tolerance were identified on Ch2 and Ch6 on the Carrizo citrange genetic map. The QTL on Ch2 in 2023, *qHLB-Cc.pau2.1*, located at 112.5 cM, had an LOD score of 3.7 and and a PVE value of 11.9%. The QTL (*qHLB-Cc.pau2.2*) on Ch2 was mapped in 2024 at 113.3 cM, having an LOD score of 3.2 with a PVE of 10.7%. Another QTL (*qHLB-Cc.pau6.1*) was identified on Ch6 in the same year, located at 65.8 cM, with an LOD score of 3.2 and a PVE of 9.7%. For the average of 2023 and 2024, two QTLs (*qHLB-Cc.pau2.3* and *qHLB-Cc.pau6.2*) were detected on Ch2 (113.3 cM) and Ch6 (60.8 cM), with LOD scores of 3.3 and 3.9, and PVEs of 10.3%, and 11.0%, respectively (**Table 5**; **Supplementary Figure 6**).

**Table 5:** Quantitative trait loci identified for HLB disease tolerance on the Carrizo citrange (♂) genetic map.

Trait name	QTL name	Year	Chr. no.	Position	LOD	PVE (%)	Marker interval	Confidence interval (cM)
HLB	*qHLB-Cc.pau2.1*	Y1	2	112.5	3.7	11.9	C2_7317358–C2_6953182	111.7–113.3
	*qHLB-Cc.pau2.2*	Y2	2	113.3	3.2	10.7	C2_7197294–C2_6866307	112.5–114.2
	*qHLB-Cc.pau6.1*	Y2	6	65.8	3.2	9.7	C6_18561999–C6_19460089	65.0–66.7
	*qHLB-Cc.pau2.3*	AVG	2	113.3	3.3	10.1	C2_7197294–C2_6866307	112.5–114.2
	*qHLB-Cc.pau6.2*	AVG	6	60.8	3.9	11.0	C6_17372960–C6_17671710	59.2–61.7
pH	*qpH-Cc.pau2.1*	Y1	2	113.3	3.8	9.8	C2_7197294–C2_6866307	112.5–114.2
	*qpH-Cc.pau6.1*	Y1	6	61.7	3.9	12.2	C6_17524952–C6_18276897	60.8–62.5
	*qpH-Cc.pau8.1*	Y1	8	65.8	4.0	2.2	C8_4301337–C8_3050151	65.0–66.7
	*qpH-Cc.pau2.2*	Y2	2	113.3	3.9	12.1	C2_7197294–C2_6866307	112.5–114.2
	*qpH-Cc.pau2.3*	AVG	2	113.3	4.0	10.1	C2_7197294–C2_6866307	112.5–114.2
	*qpH-Cc.pau6.2*	AVG	6	60.8	3.1	11.0	C6_17372960–C6_17671710	59.2–61.7
LC	*qLC-Cc.pau1.1*	Y1	1	15.8	3.0	7.5	C1_1675432–C1_12994954	15.0–16.7
	*qLC-Cc.pau7.1*	Y2	7	50.8	3.5	8.2	C7_10088679–C7_8628356	50.0–51.7
	*qLC-Cc.pau7.2*	AVG	7	50.8	3.4	7.9	C7_10088679–C7_8628356	50.0–51.7

HLB, Huanglongbing; pH, percent difference before and after infection; LC, leaf chlorophyll content; Y1, year 2023; Y2, year 2024; AVG, average from both years; LOD, logarithm of odds; PVE, phenotypic variance explained.

Six QTLs for pH were identified on chromosomes 2, 6, and 8 on the Carrizo citrange genetic map. In the year 2023, the first QTL, *qpH-Cc.pau2.1*, located at 113.3 cM, had an LOD score of 3.8 and a PVE of 9.8%. For the same year, two QTLs (*qpH-Cc.pau6.1* and *qpH-Cc.pau8.1*) were identified on Ch6 (61.7 cM) and Ch8 (65.8 cM), having LOD scores of 3.9 and 4.0 with PVEs of 12.2% and 2.2%, respectively. For the year 2024, only one QTL, *qpH-Cc.pau2.2*, was detected on Ch2 at 113.3 cM, having an LOD score of 3.9 with a PVE of 12.1%. For the average of 2023 and 2024, two QTLs, namely, *qpH-Cc.pau2.3* and *qpH-Cc.pau6.2*, were found on Ch2 (113.3 cM) and Ch6 (60.8 cM), having LOD scores of 4.0 and 3.1 with PVEs of 10.3% and 11.0%, respectively (**Table 5**; **Supplementary Figure 6**).

Three QTLs for leaf chlorophyll content were identified on the Carrizo citrange genetic map situated on Ch1 and Ch7. The QTL *qLC-Cc.pau1.1* (2023) on Ch1 mapped at 15.8 cM and had a PVE value of 7.5% with an LOD score of 3.0, while two QTLs on Ch7, *qLC-Cc.pau7.1* (2024) and *qLC-Cc.pau7.2* (AVG), mapped at the same position (50.8 cM), both showing consistent marker intervals and PVE values of 8.2% and 7.9%, with LOD scores of 3.5 and 3.4, respectively (**Table 5**; **Supplementary Figure 6**). Overall, the QTL data for the male linkage map indicate strong and consistent QTL regions for HLB and pH on Ch2 and Ch6, and for leaf chlorophyll content on Ch7, with several QTLs identified across both years.

The confidence intervals of the major QTLs on the genetic maps indicated that some QTLs remained consistent across both years and their averaged data. For the QTLs linked to PDI (HLB), three were co-localized on Ch1 and Ch6 in the “Daisy” tangerine map, while two were co-localized on Ch2 and Ch6 in the Carrizo citrange map. For QTLs linked to pH, three showed co-localization on Ch1, Ch2, and Ch6 in the “Daisy” tangerine map, whereas in the Carrizo citrange map, three were co-localized on Ch2, Ch6, and Ch8. Regarding QTLs associated with leaf chlorophyll content, two were co-localized on Ch2 and four in the “Daisy” tangerine map, while in the Carrizo citrange map, two QTLs were co-localized on Ch1 and Ch7. Notably, co-localization of QTLs across the three traits was recorded over 2 years and the AVG in the Carrizo citrange map on Ch2, Ch6, and Ch7. Similarly, co-localization of QTLs was noted on Ch1, Ch2, and Ch4 in the “Daisy” tangerine map. Overall, the positions of the QTLs linked to the three traits were grouped into five primary clusters on Ch1, Ch2, Ch4, Ch6, and Ch7. Among these clusters, QTLs on Ch1, Ch2, Ch4, and Ch6 were the most consistent, accounting for the largest portion of phenotypic variation.

## 4 Discussion

HLB is a significant challenge to citrus production worldwide, necessitating the development of sustainable and environmentally friendly control strategies. Of the various approaches, the breeding of resistant citrus varieties through MAS has emerged as the most effective method (Huang et al., 2018). MAS accelerates the breeding process by enabling the identification and incorporation of desirable phenotypic traits in a significantly shorter time. Despite the urgency of addressing HLB, there has been limited progress in understanding the genetic basis of resistance. To date, limited reports have documented the inheritance and mapping of HLB resistance (Huang et al., 2018; 2023). Moreover, no molecular marker has yet been identified that is tightly linked to traits associated with HLB tolerance.

### 4.1 Phenotypic characterization and QTL mapping

The present study is the first report from India to identify QTLs associated with tolerance to HLB disease in the Carrizo citrange × “Daisy” tangerine F_1_ hybrid population. Recent QTL studies in citrus have primarily focused on morphological and physiological characteristics (Imai et al., 2017; Yu et al., 2017; Curtolo et al., 2017; Yu et al., 2016; Raga et al., 2016; Asins et al., 2015; Raga et al., 2014; Sahin-Cevik and Moore, 2012; Raga et al., 2012; Sugiyama et al., 2011; Kepiro and Roose, 2010). Only a few studies have examined disease-associated QTLs, including resistance to ABS (Cuenca et al., 2016, 2013), CTV (Ohta et al., 2015; Asins et al., 2012), the citrus nematode (Ling et al., 2000), and CiLV (Bastianel et al., 2009). In plant genetics, the influence of QTLs on disease tolerance/resistance typically ranges from a few percent to over 50%, with QTLs having >10% of phenotypic variation categorized as major QTLs, and those accounting for over 50% considered dominant QTLs (St Clair, 2010; Davey et al., 2006). In our research, three clusters of QTLs related to HLB tolerance were detected on the genetic maps of the “Daisy” tangerine and two on the genetic map of the Carrizo citrange. Although the majority of these may be classified as major QTLs, none individually accounted for the majority of the phenotypic variation. These findings suggest that the high level of resistance/tolerance to HLB disease in Carrizo citrange is not controlled by a single gene, but rather by five genomic regions/QTLs. The mechanism of HLB pathogenesis remains poorly understood and is highly complex (Wang et al., 2017; Martinelli and Dandekar, 2017). Recent studies on HLB have proposed that citrus responses to the disease involve at least three primary molecular mechanisms, encompassing numerous genes and pathways (Martinelli and Dandekar, 2017). These findings are consistent with our results, which indicate that HLB tolerance is polygenic.

The resistance observed for diseases such as CiLV, CTV, ABS, and citrus nematode within the citrus gene pool contrasts with the response of *P. trifoliata* to HLB, which is more appropriately described as tolerance rather than resistance. While *P. trifoliata* was initially reported as resistant to *C*Las infection (Folimonova et al., 2009), recent studies suggest that it is not truly resistant but instead exhibits a high level of tolerance (Miles et al., 2017; Ramadugu et al., 2016). In this study, we used Carrizo citrange as the tolerant parent for HLB (Albrecht and Bowman, 2012; Shokrollah et al., 2011). Huang et al. (2018) observed that trifoliate oranges often tested negative for HLB, although some replicates showed marginal *C*Las diagnostic results with qPCR Ct values (33 to 39), and some of them were even HLB positive (Ct values below 33). In our research, although the Carrizo citrange was used as a tolerant parent, Ct values ranged from 18.46 to 19.02 across 2 years. Similarly, Folimonova et al. (2009) observed the lowest bacterial titer levels in Carrizo citrange and *Severinia buxifolia.* This result likely reflects the high artificial HLB inoculum pressure within the insect-proof cage. Despite *C*Las infection, Carrizo citrange displayed no HLB-associated symptoms, suggesting the presence of a tolerance mechanism in this parent.

To ensure phenotypic accuracy, we used four replicates of control budded plants for each genotype, and a total of seven genotypes/controls, throughout the phenotyping process. This robust methodology ensured reliable results, and we believe that all tested genotypes, including Carrizo citrange, were infected with *C*Las, maintaining high bacterial titers. Importantly, Carrizo citrange remained asymptomatic under intense HLB pressure, suggesting an ability to suppress *C*Las growth and maintain health even when infected. Similarly, little or no foliar disease symptoms or growth reduction were reported in Carrizo citrange by Albrecht and Bowman (2012). Previous studies have demonstrated that Carrizo citrange, US-802, and Volkamer lemon are able to restrict bacterial multiplication during the early stages of infection, although disease symptoms eventually developed under certain conditions. In the present study, Carrizo citrange did not exhibit noticeable HLB symptoms despite a detectable *C*Las infection, which may be attributed to environmental influences and variations in disease expression under the prevailing experimental conditions. Furthermore, the incidence of infection was lowest (20%–22%) in trees on Carrizo citrange, Sour orange, and Rough lemon and highest (56%–60%) in trees on US-801, US-897, and Sun Chu Sha (Albrecht et al., 2012). Among the F_1_ progenies, varying degrees of HLB tolerance were observed, with some progenies showing diagnostic and phenotypic results similar to Carrizo citrange, indicating that HLB tolerance is heritable. Similarly, hybrids of *P. trifoliata* have also demonstrated tolerance to HLB disease (Ramadugu et al., 2016; Albrecht and Bowman, 2012, 2011), likely because of the partial inheritance of HLB disease tolerance from *P. trifoliata*.

### 4.2 SNP genotyping and genetic linkage mapping

In the current study, we developed two distinct parental genetic maps, which, to the best of our knowledge, represent the highest-density genetic maps for “Daisy” tangerine and Carrizo citrange to date. The parentage of Carrizo citrange consists of a cross between *C. sinensis* “Washington” sweet orange and *P. trifoliata*. A previously reported high-density genetic map of *C. sinensis* included 799 SNPs and 189 SSRs, covering a total genetic length of 1,026.6 cM with an average marker density of 1.0 markers per cM (Lyon, 2008). Another map comprised 754 SNPs, spanning a total genetic length of 760.2 cM with an average of 1.0 marker per cM (Huang et al., 2018). The previously reported high-density genetic map of *P. trifoliata* contained 146 SNP markers and 74 SSR markers, spanning a total genetic length of 937.1 cM and having an average density of 0.2 marker/cM (Lyon, 2008), and another genetic map consisting of 2,778 DArTseq markers spanned a total genetic length of 2,446.6 cM (Curtolo et al., 2017). Additionally, Huang et al. (2018) constructed a high-density linkage map using 647 SNP markers, covering a genetic distance of 1,030.8 cM, with an average marker density of 1.6 markers per cM. The Carrizo citrange map generated in this study included 1,117 unique SNPs, covering a genetic distance of 1,095.8 cM, having an average density of 1.0 markers per cM, providing significantly higher coverage of the citrus genome compared to existing genetic maps. As a result, our genetic mapping has significantly enhanced the density of the genetic map of Carrizo citrange. This updated linkage map can serve as a reference for the future genome assembly of Carrizo citrange, as no direct map is currently available for its genome. “Daisy” tangerine is a hybrid of Fortune mandarin × Fremont mandarin. The previously reported highest density genetic maps of “Fortune” consisted of 189 SNPs, covering a genetic length of 681.1 cM and with an average density of 4.1 markers per cM (Yu et al., 2016), while another map of “Murcott” consisted of 106 SNP markers, spanning 395.3 cM and having an average density of 4.3 markers per cM (Yu et al., 2016). We developed a high-density genetic map for “Daisy” tangerine, incorporating 788 SNPs that cover a total genetic length of 995.8 cM, having an average marker density of 1.2 markers per cM. This enhancement is likely due to the superior quality of genotyping and the refined linkage mapping approach used in the construction of the map.

Developing high-resolution genetic maps for citrus remains more challenging compared to model species, which typically feature thousands of molecular markers with greater accuracy and precision (Ollitrault et al., 2012a). Despite primarily identifying a large number of SNPs, only a small number of SNPs could be successfully mapped onto different linkage groups. This challenge has been frequently noted in earlier SNP marker-based genetic maps of citrus, where numerous markers tend to group together in regions exhibiting “zero recombination”, signifying limited meiotic recombination among markers in these segments (Imai et al., 2017; Curtolo et al., 2017; Yu et al., 2016; Guo et al., 2015; Shimada et al., 2014; Lyon, 2008). Such marker redundancy is often attributed to factors such as small population sizes, closely spaced physical markers, and low-recombination regions. Increasing population size and achieving a more uniform marker distribution are commonly suggested solutions to these challenges in many plant species; however, applying these methods to citrus is particularly difficult (Curtolo et al., 2017; Imai et al., 2017; Yu et al., 2016; Guo et al., 2015). Citrus plants exhibit biological characteristics such as seedlessness, prolonged juvenility, polyembryony, high levels of heterozygosity, apomixis, gametophytic self-incompatibility, gametic, and zygotic selection (Gaikwad et al., 2026a, b, c; Gaikwad et al., 2025; Sidhu et al., 2024a, b; Gaikwad et al., 2024; Shimada et al., 2014). These traits pose challenges in the development of distinctive segregating markers and the establishment of extensive full-sib populations while also significantly influencing allelic segregation and RFs (Ollitrault et al., 2012a; Bernet et al., 2010).

In citrus hybridization studies, the genotypes and the homology of the parent plants significantly influence the density of genetic maps. For instance, Guo et al. (2015) constructed a comprehensive genetic map of pummelo using 1,543 SNPs in a full-sib and intraspecific F_1_ population comprising 124 hybrid progeny. In another study, Curtolo et al. (2017) developed a genetic map using a full-sib and interspecific F_1_ population of 278 plants generated from a cross between a tangor and a sweet orange. However, the authors were only able to map 661 SNP-based DArTseq markers onto the integrated map. This comparison highlights that intraspecific populations tend to produce genetic maps with much higher marker densities than the interspecific populations, even though the size of the population was considerably smaller (Huang et al., 2018).

In the current study, the use of intergeneric hybrids in the mapping population posed additional challenges for the construction of higher-density genetic maps. Beyond the influence of parental genotype, factors such as crossing direction, the differential fitness of gamete genotypes, and interactions between regulatory genes probably contributed to the significant segregation distortion observed in the citrus species (Curtolo et al., 2017; Bernet et al., 2010). Excluding polymorphic/potential loci significantly decreased the number of segregating SNPs, particularly in the “Daisy” tangerine map, and resulted in extensive regions on the genetic maps with a low marker density. Moreover, the “Daisy” tangerine genetic map contained a significantly lower number of segregating markers compared to the Carrizo citrange map. This issue has been consistently reported in earlier citrus genetic maps (Ollitrault et al., 2012a; Lyon, 2008; Chen et al., 2008). The reduced number of segregating markers in trifoliate orange is primarily attributed to its low level of heterozygosity (Huang et al., 2018). However, in the current study, Carrizo citrange exhibited a higher number of segregating markers than “Daisy” tangerine.

Significant variations in chromosome sizes were recorded between the genetic maps of Carrizo citrange and “Daisy” tangerine. The Carrizo citrange map had a longer genetic length compared to the “Daisy” tangerine map, despite having a higher number of mapped markers. Similar discrepancies in genetic distances have been reported in earlier EST-SSR-based genetic maps constructed using codominant markers that segregated in both parents (Huang et al., 2018; Chen et al., 2008). Variations in map lengths have also been documented in SNP-based genetic maps of sweet orange, pummelo, and mandarin (Huang et al., 2018; Ollitrault et al., 2012a; Chen et al., 2008). With the integration of reference genomes, SNP-based genetic linkage maps can be compared relative to physical distances. However, differences in genetic map lengths are not linked to genome size, which ranges from 360 to 398 Mb across citrus species (Huang et al., 2018; Wu et al., 2018, 2014; Gmitter et al., 2012; Chen et al., 2008). Nonetheless, simulation studies suggest that gametic selection-induced segregation distortion has a minimal impact on marker order and genetic distances (Hackett and Broadfoot, 2003). Therefore, the differences in genetic map lengths between Carrizo citrange and “Daisy” tangerine are likely to be due to differential recombination rates rather than segregation distortion. Research in model plants has shown that genome sequence divergence can significantly affect recombination rates, with lower recombination rates being associated with higher genome divergence (Li et al., 2006; Opperman et al., 2004; Chetelat et al., 2000). Among citrus-related genera and species, heterozygosity levels vary substantially. For instance, mandarins are highly heterozygous (Wang et al., 2018), whereas trifoliate orange, a genus that is related to *Citrus*, exhibits significantly lower heterozygosity (Huang et al., 2018; Chen and Gmitter, 2013; Ollitrault et al., 2012a; Chen et al., 2008).

Compared to Carrizo citrange, the higher heterozygosity of the “Daisy” tangerine genome likely reduces RF, leading to a smaller genetic size/map. This aligns with the observed differences in genetic distance among shared markers on the genetic maps of trifoliate orange and sweet orange (Huang et al., 2018), in addition to pummelo and Clementine mandarin (Ollitrault et al., 2012a). Carrizo citrange is a hybrid of *C. sinensis* “Washington” sweet orange and *P. trifoliata*, while “Daisy” tangerine is derived from a cross between Fortune mandarin (*Clementine mandarin × Orlando tangelo*) and Fremont mandarin (*Clementine mandarin × Ponkan mandarin*). Clementine mandarin is a hybrid (interspecific) of *C. reticulata* and *C. sinensis* and is characterized by significant genome heterozygosity (Wu et al., 2014), in contrast to pummelo, one of the ancestral species of *Citrus*, which displays low genome heterozygosity (Wang et al., 2017). The predominantly heterozygous nature of EST-SSR loci in sweet orange, contrasted with their homozygosity in *Poncirus*, complicates achieving balanced genome coverage. Similarly, in our study, the Carrizo citrange map showed higher homozygosity compared to the more heterozygous “Daisy” tangerine map. Consequently, more SNPs are required to obtain enough segregating loci to achieve comparable map coverage (Chen et al., 2008). Additionally, RFs vary among sexes in both animals and plants (Lorch, 2005). In our research, the male linkage map (Carrizo citrange) was slightly larger than the female linkage map (“Daisy” tangerine) with lengths of 1,095.8 and 995.8 cM, respectively. This contrasts with Clementine mandarins, where the male genetic map is considerably shorter than the female map (Yu et al., 2016).

### 4.3 QTL mapping associated with HLB tolerance

A comparative analysis of the present study with findings from Huang et al. revealed that both conserved and unique genomic regions are associated with HLB tolerance-related traits in citrus. Using the “Daisy” tangerine × Carrizo citrange mapping population in the present investigation, several QTLs associated with HLB disease severity, pH, and leaf chlorophyll content (LC) were identified on chromosomes 1, 2, 4, 6, 7, and 8, with a PVE of up to 12.2%. Major and stable QTLs were repeatedly detected on chromosomes 2 and 6, both across individual years and their average, indicating the significance of these genomic regions in regulating HLB tolerance mechanisms. Similar observations were reported by Huang et al. (2018), who detected QTLs for foliar symptoms and canopy damage separately on trifoliate orange and sweet orange genetic maps, particularly on linkage groups t6, t8, t9, and s7. The QTLs identified on linkage groups t6 and t8 showed relatively high LOD scores (up to 5.5) and explained 13.9%–29.9% of phenotypic variation, suggesting the presence of major-effect loci contributing to HLB response. Notably, recurrent QTLs detected on linkage group t6 for foliar symptoms and canopy damage (Huang et al., 2018) correspond well with the stable genomic regions identified on chromosome 6 in the present study, supporting the importance of this chromosome in defense responses against HLB infection in citrus. Similarly, QTLs identified on linkage groups t8 and t9 overlapped with regions associated with physiological traits and disease severity observed in the current study, indicating the possible conservation of HLB-responsive loci across different citrus genetic backgrounds (Huang et al., 2018).

Furthermore, Huang et al. (2023) expanded these findings by identifying additional QTLs associated with ACP titer, healthy tree percentage, canopy volume, trunk girth, trunk growth rate, leaf symptoms, and canopy damage on trifoliate orange linkage groups t6, t7, t8, and t9. Several QTLs reported in that study exhibited high PVE values, ranging from 13.0% to 26.1%, particularly for leaf symptoms and trunk growth-related traits, highlighting the substantial contribution of trifoliate orange-derived alleles to HLB tolerance. The repeated identification of QTLs on linkage groups t6 and t8 in both studies (Huang et al., 2018, 2023) and the present study strongly suggests that these genomic regions harbor key genes involved in HLB tolerance, disease symptom suppression, and the maintenance of tree vigor under infection pressure. However, the present study also identified additional QTLs on chromosomes 1, 2, and 4 that had not been previously reported, indicating the possibility of novel loci specific to the “Daisy” tangerine × Carrizo citrange population, or environmental interactions influencing QTL expression. Variations among studies in QTL position, LOD score, and PVE may be attributed to differences in population structure, marker density, phenotyping methods, environmental conditions, and disease pressure. Nevertheless, the consistency of several major QTL regions across independent studies enhances the reliability of these loci and underscores their potential for use in MAS, fine mapping, and pyramiding of durable HLB tolerance genes in citrus breeding programs.

## 5 Conclusion

This study utilized a genotyping-by-sequencing (GBS) approach (ddRAD sequencing) on an intergeneric F_1_ population of 120 seedlings derived from the cross between Carrizo citrange and “Daisy” tangerine to construct high-density genetic maps for the parental genotypes separately. The genetic maps revealed nine chromosomes for both parents. HLB disease evaluation across two environments, “Daisy” tangerine and Carrizo citrange, demonstrated significant differences in their responses to *C*Las infection and HLB-related traits, including disease severity, pH difference, and chlorophyll content. The offspring exhibited a distinct continuous distribution across three phenotypic traits. Five main clusters of QTLs associated with HLB-related traits were identified. These QTLs were located on Ch2 and Ch6 of the Carrizo citrange map and Ch1 and Ch6 of the “Daisy” tangerine map. To our knowledge, these QTLs collectively account for a substantial portion of the phenotypic variation associated with HLB response. The findings indicate that the response to HLB in citrus is governed by multiple QTLs. This primary work establishes a foundation for future research in India aimed at uncovering the genetic basis of HLB tolerance. Further validation of these QTLs is essential to support breeding programs aimed at enhancing tolerance to HLB disease. Additionally, refining the associated genomic regions will be critical for identifying and characterizing candidate genes involved in host responses to HLB disease. Ultimately, these identified QTLs could serve as valuable targets in citrus breeding programs to develop long-term strategies against HLB.

## Data Availability

The datasets presented in this study can be found in online repositories. The names of the repository/repositories and accession number(s) can be found below: https://www.ncbi.nlm.nih.gov/, PRJNA1163051.
