# Supplementary material for: High-density genetic mapping reveals QTLs associated with Huanglongbing tolerance in citrus
Source: Front Plant Sci. 2026 Jul 8;17:1848541. doi: 10.3389/fpls.2026.1848541 (PMC13388408; doi:10.3389/fpls.2026.1848541)
Supplement: Supplementary file 1 [file DataSheet1.docx]

**Supplementary File S1** *C*Las specific gene-based primers with their annealing temperature and reference

| **Primer Name** | **Forward Sequence** | **Reverse Sequence** | **Annealing temperature (ºC)** | **Amplicon Size** | **Reference** |
| --- | --- | --- | --- | --- | --- |
| CGB (F)  CGB (R) | TGGGTGGTTTACCATTCAGTG | CGCGACTTCGCAACCCATTG | 60 | 448 | Datar et al., (2014) |
| rplJ/rplK (F) rplJ/rplK (R) | CGTCTCGTCAAGATTGCTATCCGT | TTAAGGACGCCCTTCTCTACAACC | 55 | 180 | Ananthakrishnan et al., (2013) |

| 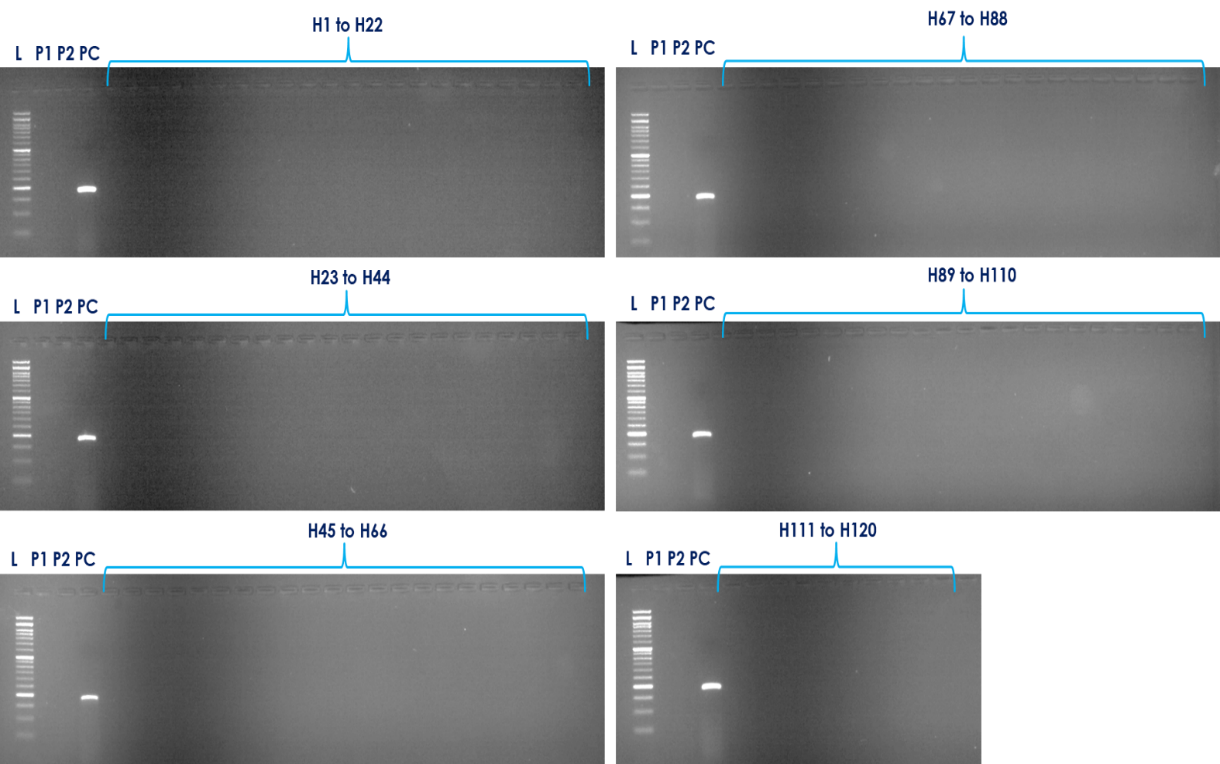 |
| --- |
| **(A)** |
| 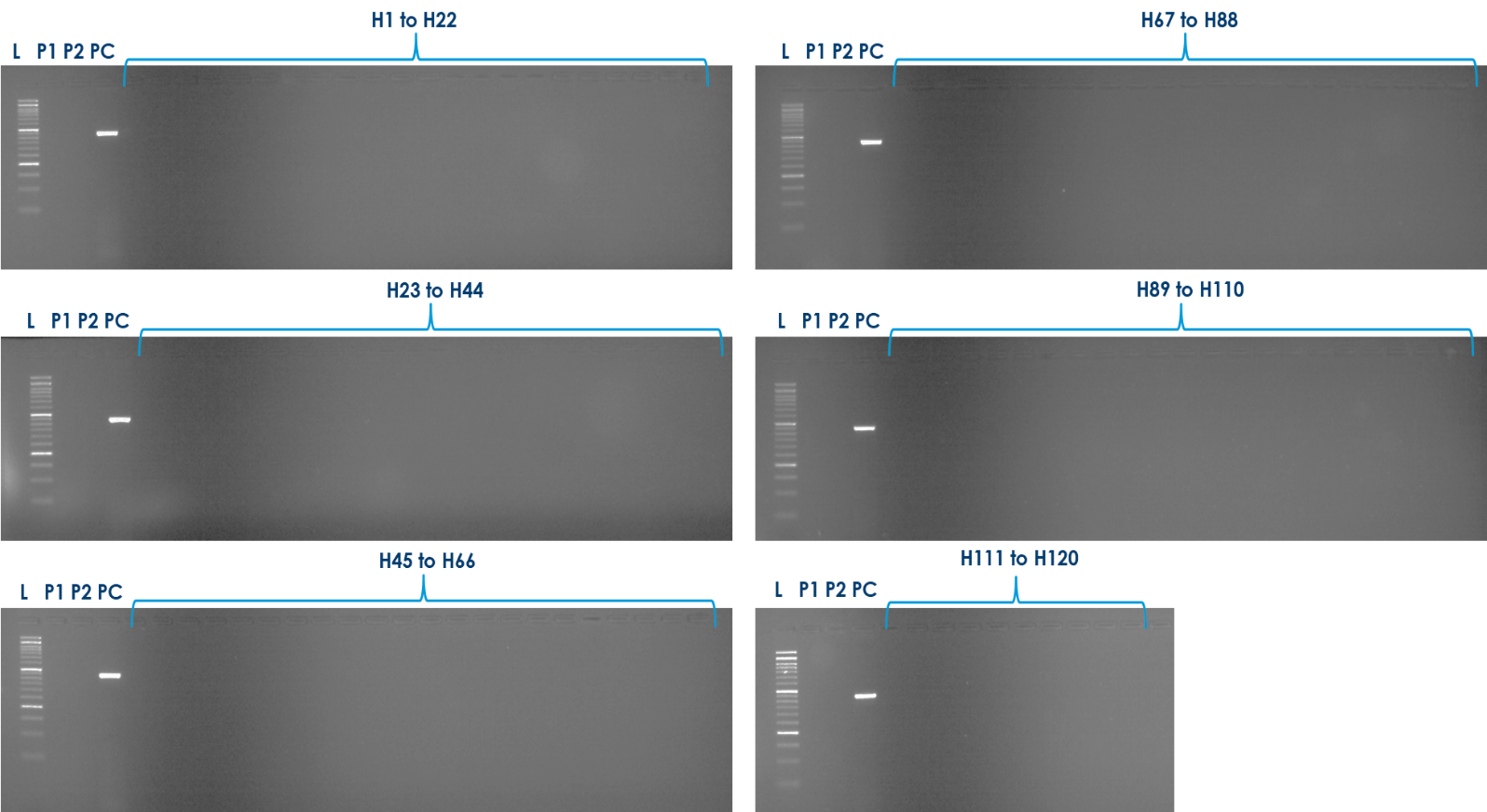 |
| **(B)** |
| **Supplementary Figure S1** Confirmation of 120 F_1_ hybrids before screening through PCR amplification of rplJ/rplK **(A)** and CGB **(B)** primer for *C*Las detection (L: 50 bp Ladder, P1: male parent (Carrizo citrange), P2: Female parent (‘Daisy’ tangerine), PC: Positive control, H: Hybrid number) |

| 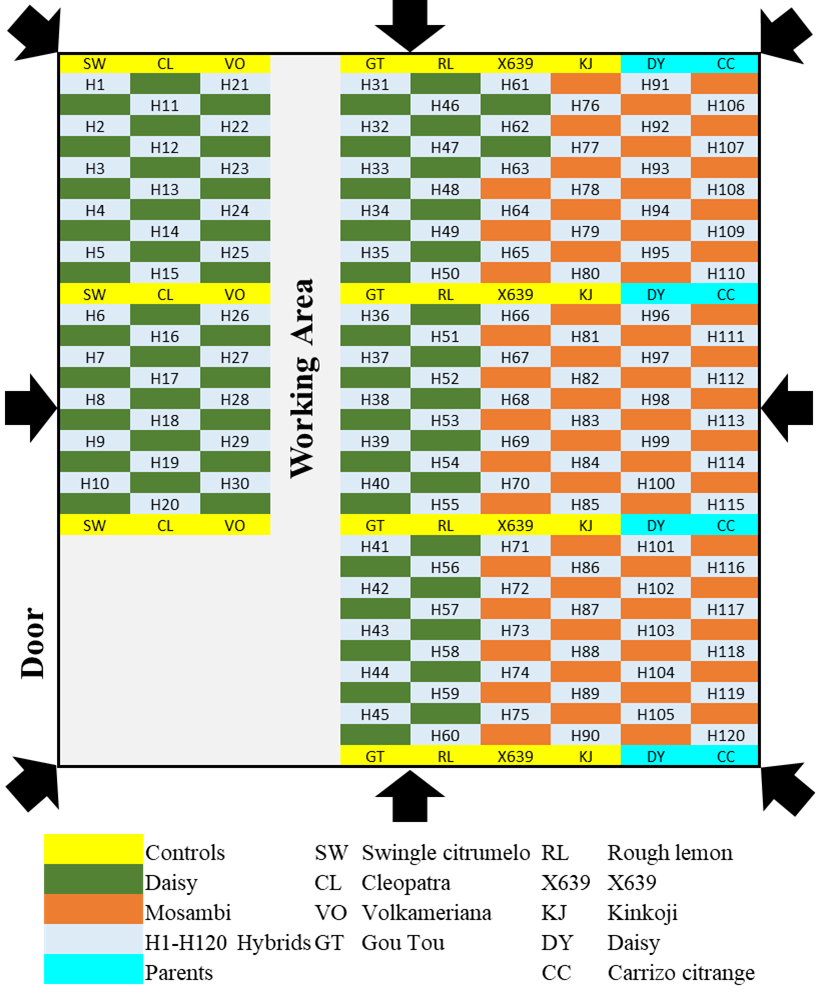 |
| --- |
| **Supplementary Figure S2** Strategy for insect release for the screening of HLB disease (solid black arrow indicates sites for insect release) |

| 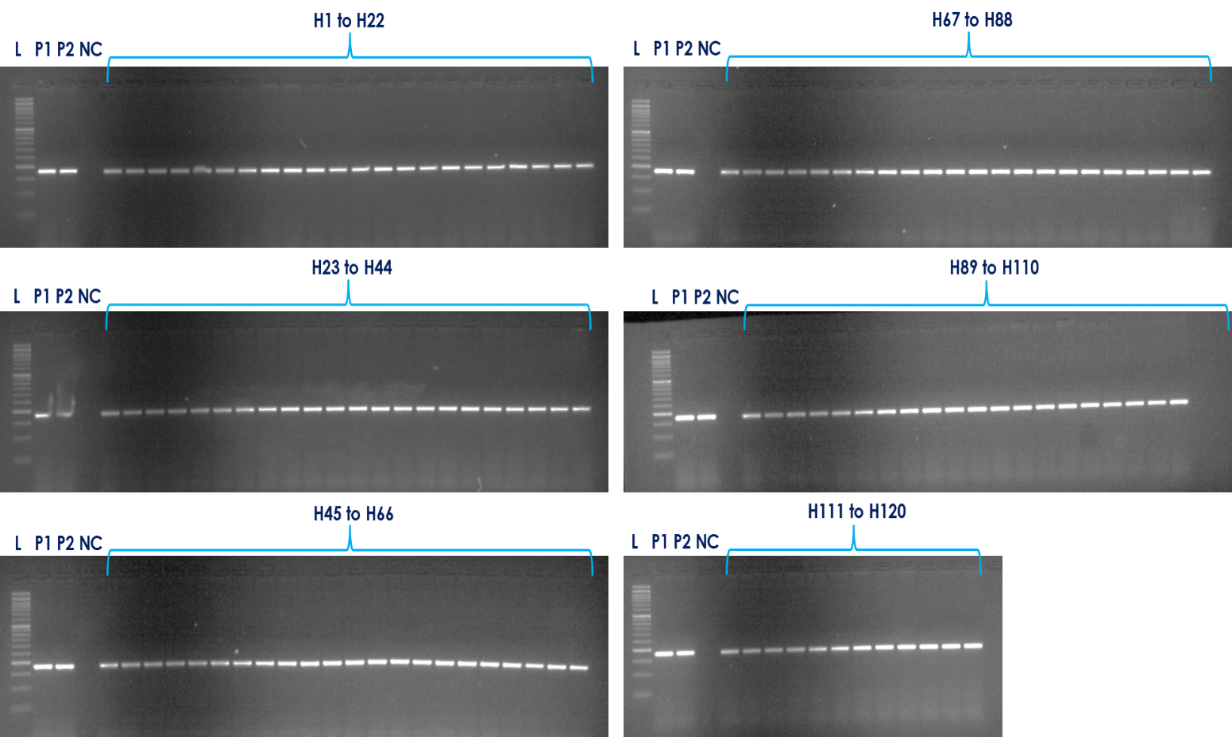 |
| --- |
| **(A)** |
| 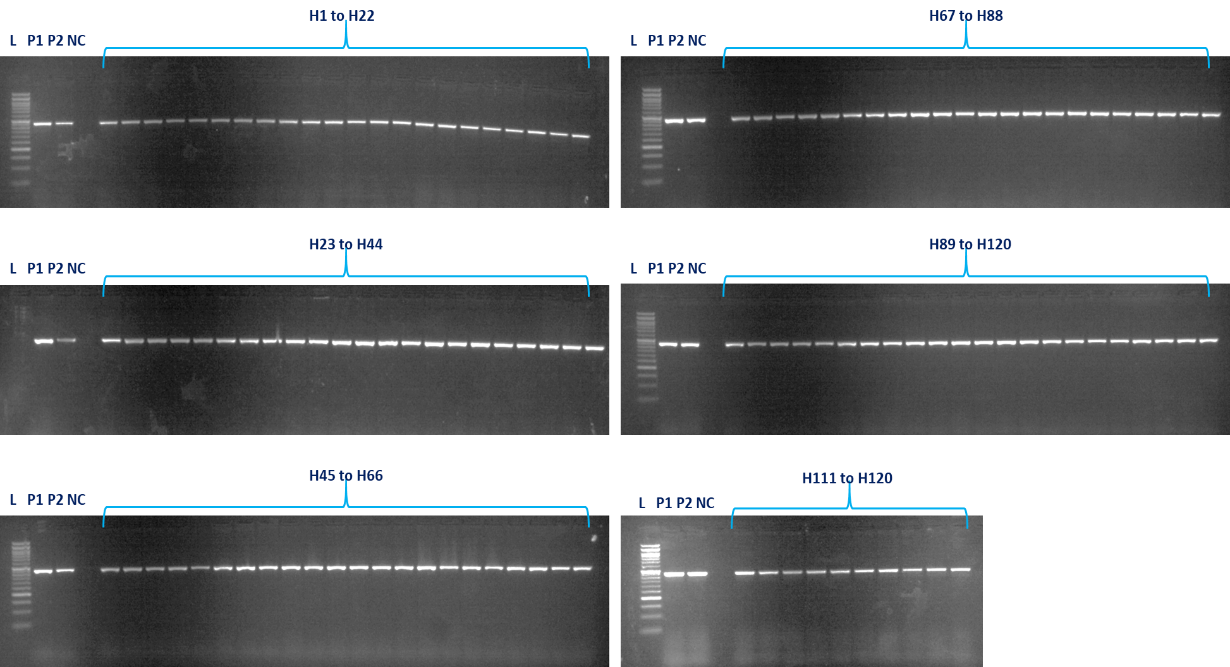 |
| **(B)** |
| **Supplementary Figure S3** Confirmation of 120 F_1_ hybrids after screening through PCR amplification of rplJ/rplK **(A)** and CGB **(B)** primer for *C*Las detection (L: 50 bp Ladder, P1: male parent (Carrizo citrange), P2: Female parent (‘Daisy’ tangerine), NC: Negative control, H: Hybrid number) |

| 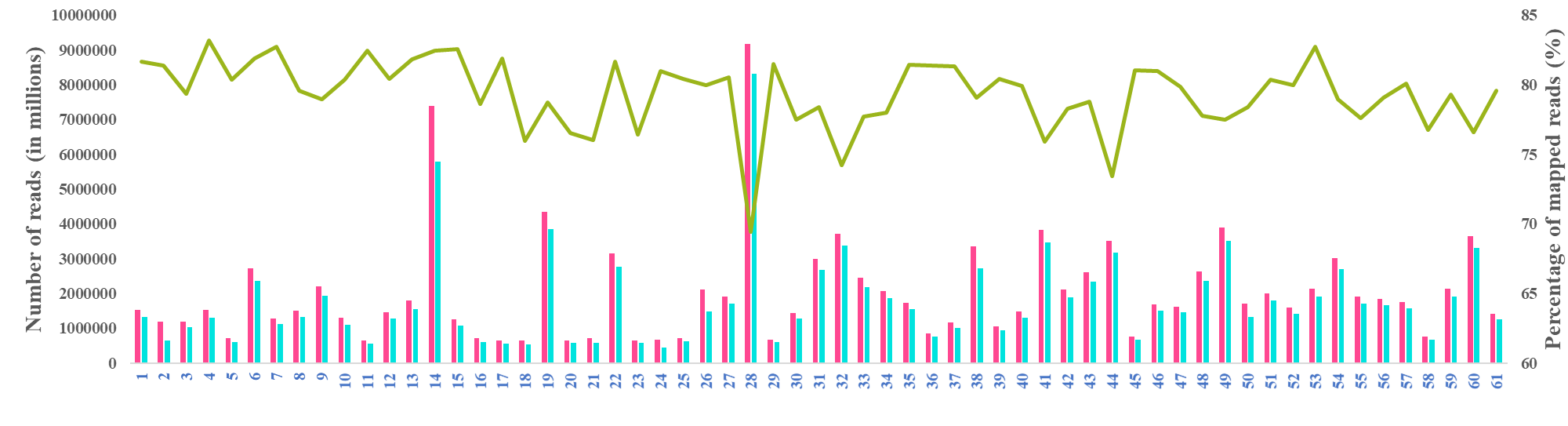 |
| --- |
| 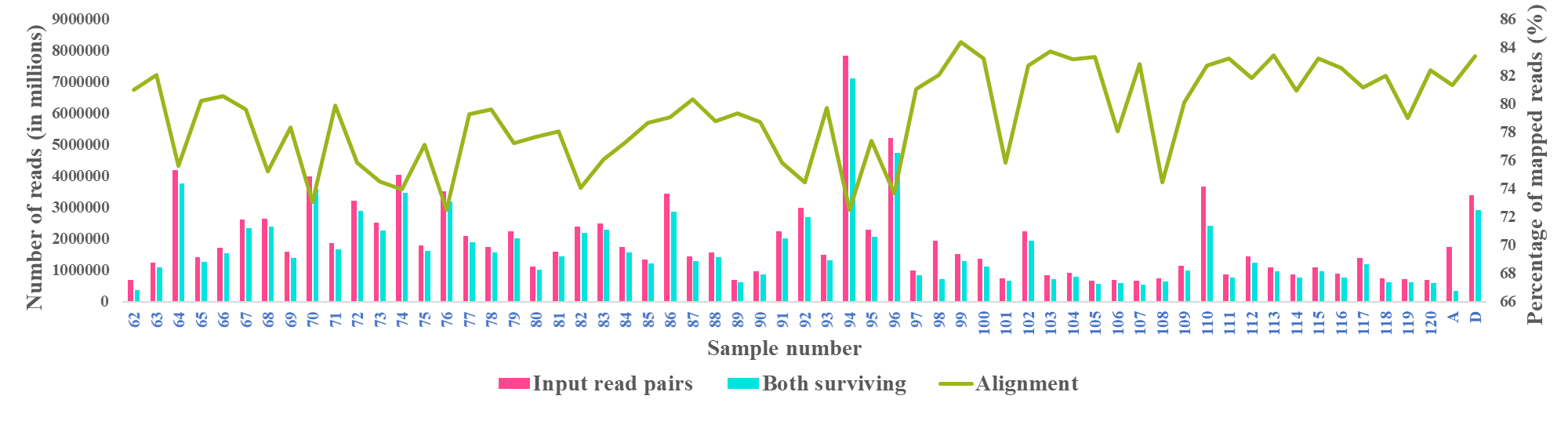 |
| **Supplementary Figure S4** Details of number of reads before and after cleaning along with percentage of mapped reads |


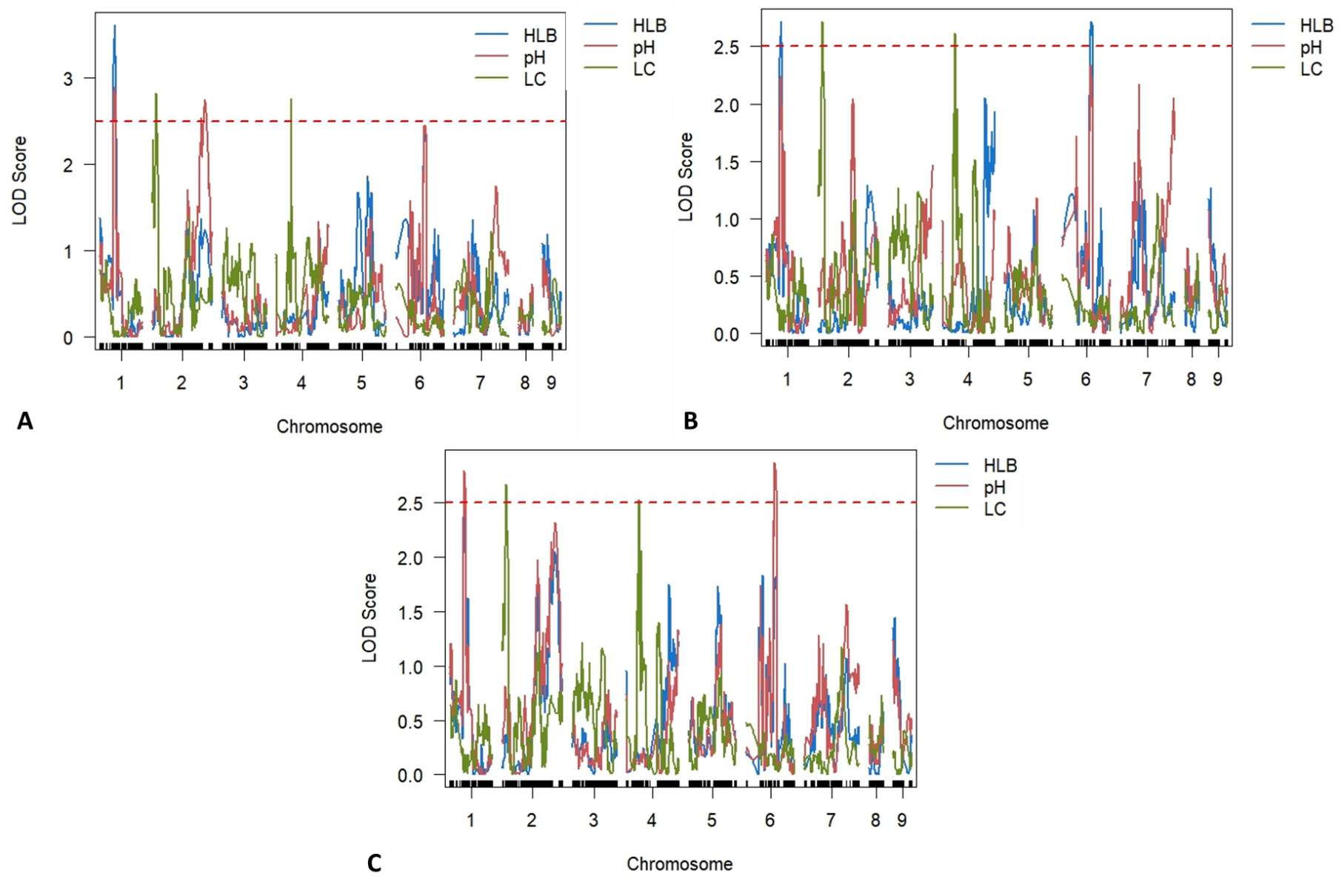


**Supplementary Figure S5** Quantitative trait locus detected for HLB disease resistance on female (‘Daisy’ tangerine) linkage map **(A)** HLB-2023, pH-2023 and LC-2023, **(B)** HLB-2024, pH-2024 and LC-2024, **(C)** HLB-Average of both years, pH-Average of both years and LC-Average of both years**.** Plots indicate the genetic coordinate (chromosome) on the x-axis and LOD score on the y-axis of detected QTLs.


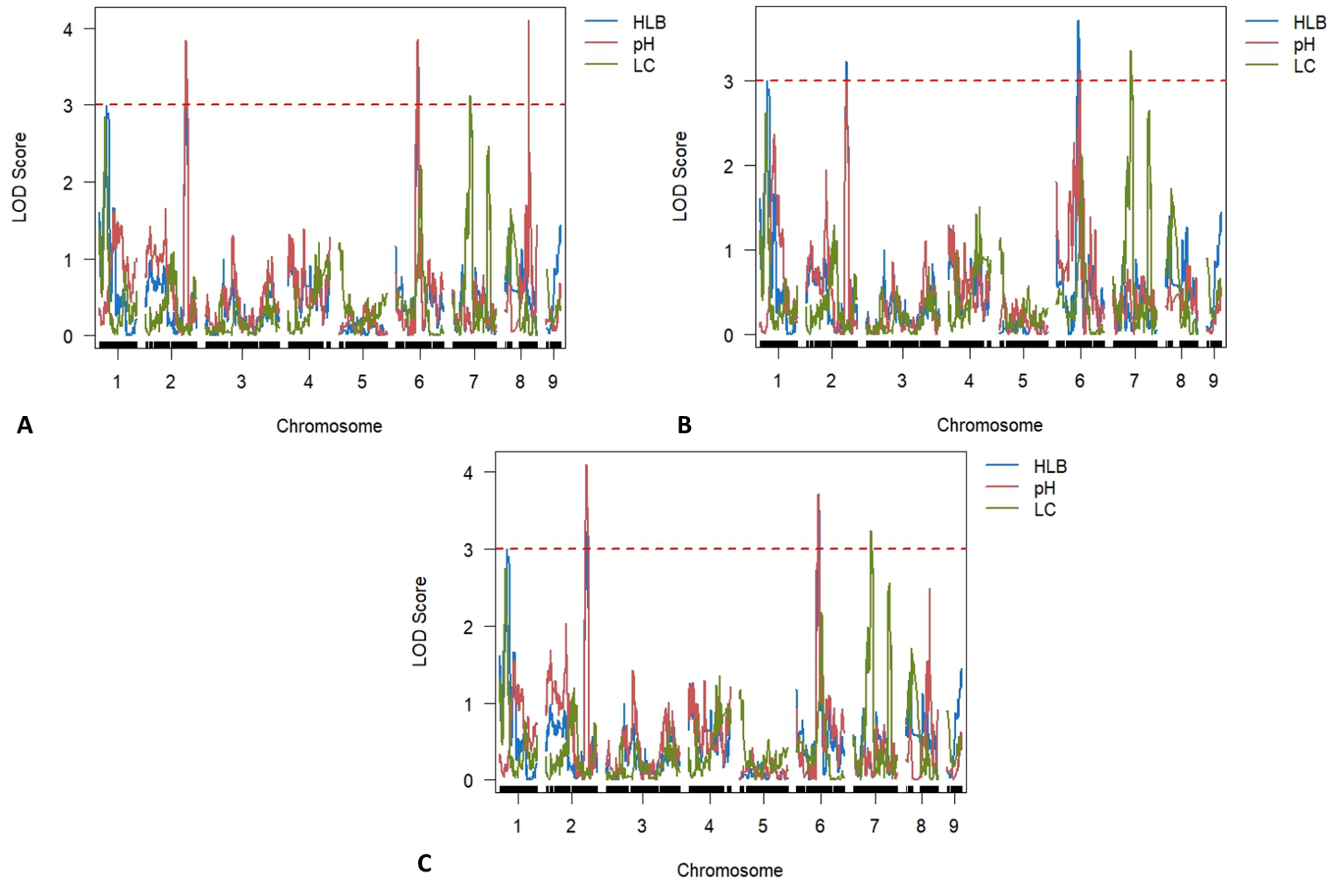


**Supplementary Figure S6** Quantitative trait locus detected for HLB disease resistance on male (Carrizo citrange) linkage map **(A)** HLB-2023, pH-2023 and LC-2023, **(B)** HLB-2024, pH-2024 and LC-2024, **(C)** HLB-Average of both years, pH-Average of both years and LC-Average of both years**.** Plots indicate the genetic coordinate (chromosome) on the x-axis and LOD score on the y-axis of detected QTLs.
